# Dental form and function in the early feeding diversification of dinosaurs

**DOI:** 10.1126/sciadv.abq5201

**Published:** 2022-12-16

**Authors:** Antonio Ballell, Michael J. Benton, Emily J. Rayfield

**Affiliations:** Bristol Palaeobiology Group, School of Earth Sciences, University of Bristol, Life Sciences Building, Tyndall Avenue, Bristol BS8 1TQ, UK.

## Abstract

Dinosaurs evolved a remarkable diversity of dietary adaptations throughout the Mesozoic, but the origins of different feeding modes are uncertain, especially the multiple origins of herbivory. Feeding habits of early dinosaurs have mostly been inferred from qualitative comparisons of dental morphology with extant analogs. Here, we use biomechanical and morphometric methods to investigate the dental morphofunctional diversity of early dinosaurs in comparison with extant squamates and crocodylians and predict their diets using machine learning classification models. Early saurischians/theropods are consistently classified as carnivores. Sauropodomorphs underwent a dietary shift from faunivory to herbivory, experimenting with diverse diets during the Triassic and Early Jurassic, and early ornithischians were likely omnivores. Obligate herbivory was a late evolutionary innovation in both clades. Carnivory is the most plausible ancestral diet of dinosaurs, but omnivory is equally likely under certain phylogenetic scenarios. This early dietary diversity was fundamental in the rise of dinosaurs to ecological dominance.

## Teaser

Early dinosaurs evolved diverse tooth shapes and mechanics as adaptations to different diets, so ensuring their success.

## INTRODUCTION

**Fig. 1.**
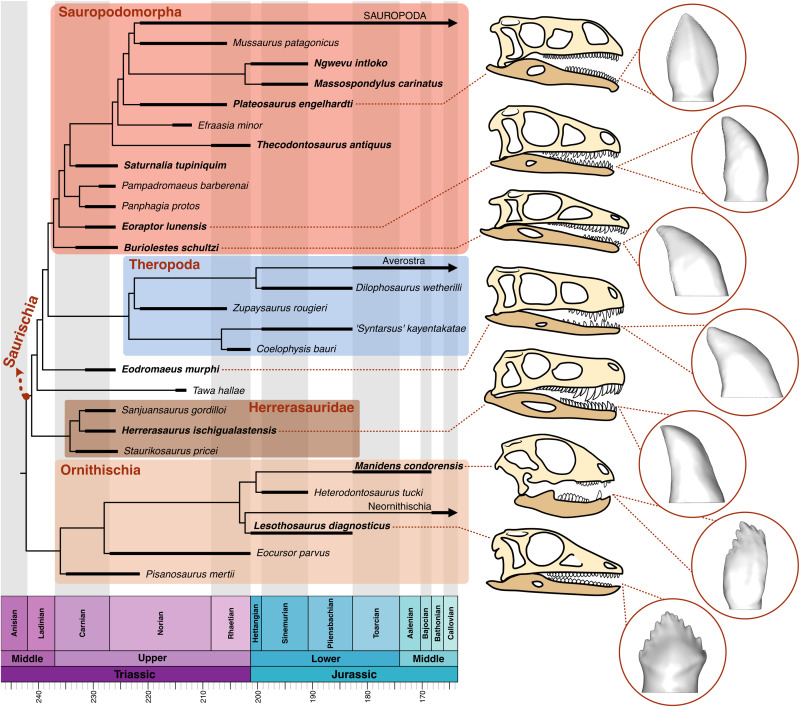
Time-calibrated phylogeny of Dinosauria including species from the Late Triassic and Early Jurassic. Names of dinosaur species in this study are marked in bold. 3D tooth models of selected dinosaurs are shown in labial view. Simplified phylogenetic tree modified from the work of Müller and Garcia (*9*) and Pol *et al.* (*86*).

Dinosaurs were an outstandingly diverse clade of archosaurs that evolved a wide range of craniodental morphologies throughout the Mesozoic (Fig. 1), implying the exploitation of varied feeding strategies and food resource use (*1*–*6*). The ancestral diet of dinosaurs is a matter of debate, partly because of alternative hypotheses for the basal topology of dinosaur phylogeny (*7*–*9*) and partly because of the rarity of direct evidence of feeding behavior (e.g., stomach contents, coprolites, etc.). Therefore, most dietary inferences have been based on observations of craniodental morphology in early dinosaurs, with a particular focus on teeth (*2*, *10*, *11*). The curved, finely serrated teeth typically seen in early saurischians and theropods (*12*, *13*) are considered indicators of carnivory, while the denticulated, lanceolate teeth of sauropodomorphs (*14*) and the triangular teeth of ornithischians (*15*) were traditionally associated with herbivorous habits. These observations formed the basis for the idea that the ancestral diet of dinosaurs was carnivory, and herbivory evolved independently at the origin of sauropodomorphs and ornithischians (*16*). This simplistic scenario has been challenged recently by the discovery of new taxa with intermediate craniodental traits (*11*, *17*–*20*), comparisons with extant analogs (*10*), and the diet and phylogenetic position of dinosauromorph clades such as silesaurids (*9*, *11*, *21*). Thus, the diversity of feeding modes in early dinosaurs and their contributions to the radiation of this successful clade remain obscure.

Tooth shape determines the ability of the feeding apparatus to break down different food items and thus obtain nutrients and energy (*22*), and this is subject to strong selective pressures. Consequently, the evolution of diverse dental morphologies in a clade can reflect the adaptation to a wide variety of diets and feeding strategies (*23*, *24*), although historical and developmental factors can impose constraints on this process (*25*). Modern quantitative techniques can characterize different functional aspects of the dentition, such as morphological diversity, complexity, and mechanics. These are powerful tools to unravel the relationship between dental form and function and support dietary inferences in extinct taxa, especially when informed by ecological information from modern analogs (*26*, *27*). These morphofunctional and ecological data, combined with machine learning classification methods, can generate robust predictions of ecology in extinct species (*28*, *29*).

Here, we investigate the dental form and function in 11 early-diverging dinosaurs, including ornithischians, sauropodomorphs, and early saurischians/theropods (Fig. 1), in comparison with 47 extant sauropsids (squamates and crocodylians) that exhibit different dietary habits. We analyze tooth three-dimensional (3D) models using finite element analysis (FEA) and landmark-based geometric morphometrics (GMM) to quantify variation in dental stress and shape among living sauropsids and ascertain where dinosaur dentitions fit in comparison with their modern analogs. We assess the relationship between dental morphology and mechanical behavior and the influence of diet on both aspects. Last, we use machine learning classification to predict the diets of early dinosaurs based on dental form and function. Our findings indicate that early dinosaurs had morphofunctionally diverse dentitions and explored a wide range of tooth shapes and mechanical behaviors occupied by extant sauropsids with different diets. A dietary shift from carnivory to herbivory through an omnivorous phase occurred in Sauropodomorpha, and early ornithischians might have exploited omnivorous diets. These results suggest an early diversification of feeding modes in Dinosauria during the Late Triassic and Early Jurassic, thereby contributing to the success of dinosaurs at a time of rapidly changing climates and vegetation.

## RESULTS

### Dental stress distribution and magnitude

**Fig. 2.**
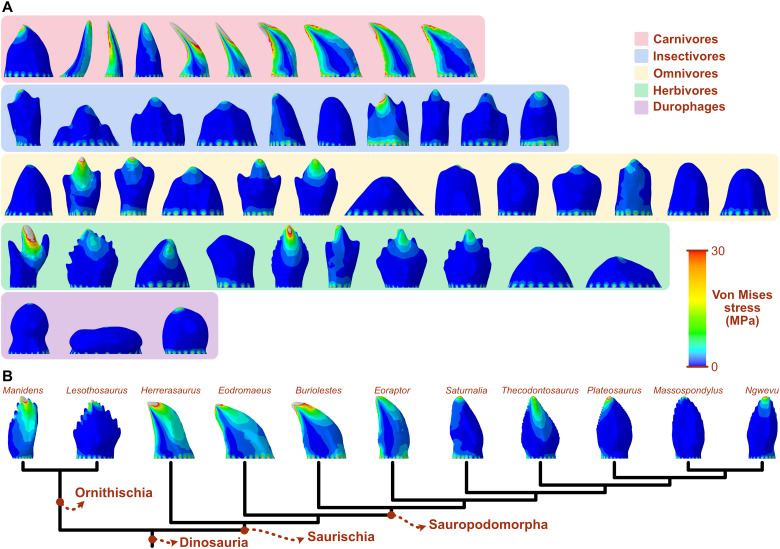
Von Mises stress distributions on the teeth of extant sauropsids and early dinosaurs. (**A**) Extant sauropsids are divided into dietary categories. (**B**) Dinosaurs are represented in a simplified phylogenetic tree modified from the work of Müller and Garcia (*9*).

The distribution of von Mises stress under a simulated biting load reflects the relative biomechanical behavior and strength of the studied tooth morphologies (Fig. 2). The teeth of most carnivorous taxa experience bending, as reflected by the cantilever-like pattern of stress distribution, with the mesial and distal cutting edges showing the highest stress. Teeth of other dietary groups experience generalized low stress with patterns consistent with compression. Herbivores show more variation in stress distribution patterns, those with simpler teeth having generally larger areas of low stress. Of the dinosaur sample, those with curved teeth, such as *Herrerasaurus*, *Eodromaeus*, *Buriolestes*, and *Eoraptor*, show larger areas of high stress and a bending-like distribution. The apicobasally high and slightly curved tooth of *Manidens* also shows high stress. *Lesothosaurus* has the largest areas of low stress, followed by *Saturnalia* and the post-Carnian sauropodomorphs.

**Fig. 3.**
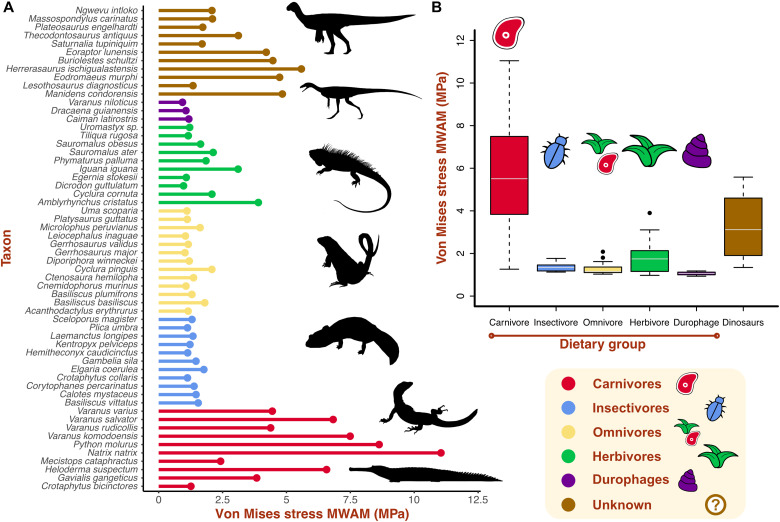
Average stress values of the teeth of extant sauropsids and early dinosaurs. (**A**) Von Mises stress MWAMs of each taxon divided by dietary category. (**B**) Von Mises stress MWAM median and range per dietary categories. Silhouettes: *Thecodontosaurus* by G. Ugueto and others from PhyloPic (http://phylopic.org) by A. Reindl, S. Traver, N. Claunch, J. M. Wood, and S. Hartman.

The mesh-weighted arithmetic mean (MWAM) (*30*) represents the average von Mises stress value of a model corrected for mesh heterogeneity (Fig. 3A). Von Mises stress mean values are substantially higher in carnivores than in the other groups, and they show the largest range of variation (Fig. 3, A and B). Omnivores and insectivores have generally low stress magnitudes, and durophages show the lowest values. Herbivores show varied mean stress values, intermediate between carnivores and the other dietary groups. Dinosaur taxa show high variation in mean tooth stress. The dinosaurs with the highest stress values are *Herrerasaurus*, *Manidens*, *Eodromaeus*, *Buriolestes*, and *Eoraptor*. The lowest stress value is recovered for *Lesothosaurus* (Fig. 3A), followed by *Saturnalia* and the plateosaurian sauropodomorphs. *Thecodontosaurus* shows an intermediate von Mises stress value.

### Biomechanical performance variation

**Fig. 4.**
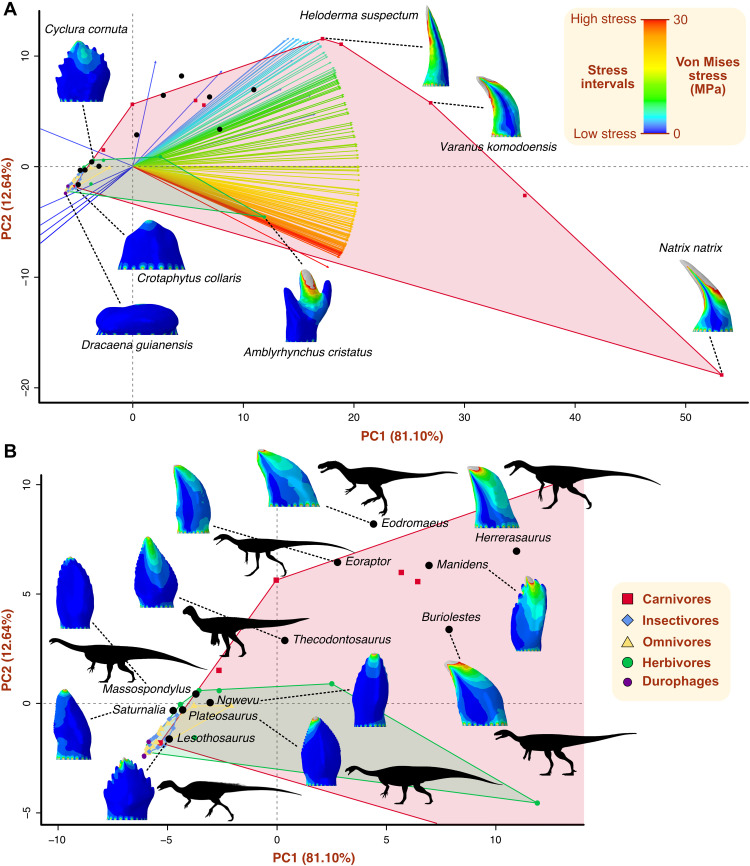
Biomechanical space representing the variation in tooth stress. (**A**) Complete dental biomechanical space captured by PC1 and PC2 axes and showing convex hulls by dietary categories. Interval variables are represented by arrows with colors that indicate stress levels. Stress distribution plots of selected taxa are shown to visualize differences in stress distribution and magnitude in biomechanical space. (**B**) Biomechanical space region occupied by early dinosaurs, accompanied by stress distribution plots. Silhouettes: *Thecodontosaurus* by G. Ugueto, *Buriolestes* by Maurissauro, *Eodromaeus* by Conty (from PhyloPic; http://phylopic.org), and others by S. Hartman (from PhyloPic).

The principal components analysis (PCA) of the biomechanical data expressed in 150 intervals decomposes the stress distribution variation into 57 principal components (PCs). The first two PC axes capture most of the stress variation (93.7%), the first representing 81.1% of the variance (Fig. 4A and fig. S4). Variation in stress variables is closely linked to PC1, with high stress variables oriented toward positive PC1 and low stress variables toward negative values of the axis (Fig. 4A). Besides their different positions in the biomechanical space (Fig. 4B), dietary groups show significantly different levels of biomechanical disparity, measured as convex hull volume, except for omnivores and insectivores (figs. S5 and S6). Carnivores occupy the largest area of the biomechanical space (fig. S5), particularly in the positive region of PC1. This area extends toward negative PC1 due to one taxon with low dental stress, *Crotaphytus bicinctores*. Herbivores have the second largest convex hull, ranging from negative to positive values of PC1. The other dietary categories are restricted to the quadrant of negative PC1 and PC2, largely overlapping in biomechanical space. Omnivores appear to occupy the largest area of these three groups, although not significantly different from the area of insectivores (fig. S6). Dinosaurs occupy a relatively large area in the center of the biomechanical space, overlapping the convex hulls of extant sauropsids (Fig. 4B). *Herrerasaurus*, *Manidens*, *Eodromaeus*, *Buriolestes*, and *Eoraptor* occupy the positive region of PC1, within the area of extant carnivores, which corresponds to high stress intervals. The ornithischian *Lesothosaurus* and the sauropodomorphs *Plateosaurus*, *Massospondylus*, *Ngwevu*, and *Saturnalia* are plotted in the negative PC1 area, with extant herbivores, insectivores, and omnivores. *Thecodontosaurus* occupies an intermediate position in biomechanical space, close to neutral PC1 and within the carnivorous convex hull.

The phylogenetic signal in dental stress variation is low but significant, both when tested on MWAM (*K*_mult_ = 0.561; *P* = 0.001) and stress intervals (*K*_mult_ = 0.524; *P* = 0.01). When *K*_mult_ is lower than 1, taxa resemble each other less than expected given their phylogenetic proximity. The effect of allometry is not significant in structuring dental stress variation, regardless of the stress variable (MWAM or intervals) or size proxy (centroid size, surface area, or volume) used (tables S6 to S11).

### Dental shape variation

**Fig. 5.**
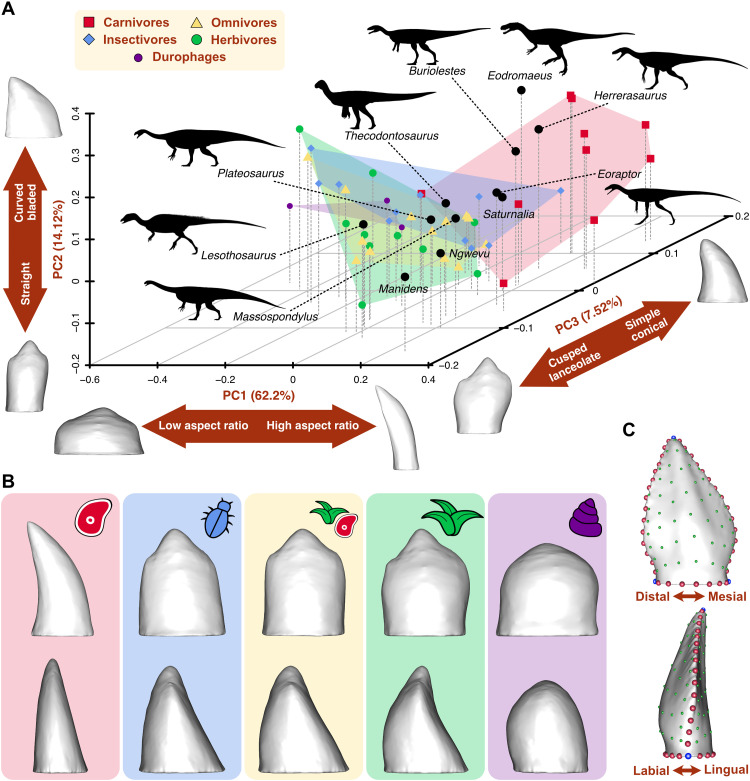
Morphological morphospace representing the variation in tooth shape. (**A**) Morphospace of the first three PC axes obtained from 3D landmark data capturing tooth morphology. Convex hulls indicate the area occupied by each dietary group. Aspects of tooth morphology explained by each PC axis are represented in double arrows, accompanied by the extreme shapes of each PC axis. (**B**) Theoretical mean shapes of each dietary category reconstructed from the Procrustes coordinates in labial (top) and mesial (bottom) views. The tooth model of the specimen closest to the Procrustes mean was warped using the mean Procrustes coordinates of each dietary group. (**C**) Landmarking protocol composed of 3 fixed landmarks (blue), 44 curve semilandmarks (red), and 98 surface semilandmarks (green), digitized on the *Thecodontosaurus antiquus* tooth model in labial (top) and mesial (bottom) views. Silhouettes: *Thecodontosaurus* by G. Ugueto, *Buriolestes* by Maurissauro, *Eodromaeus* by Conty (from PhyloPic; http://phylopic.org), and others by S. Hartman (from PhyloPic).

The morphospace resulting from the PCA performed on the Procrustes coordinates of tooth shape is represented by 57 PC axes. The first three PC axes explain 83.84% of the shape variance in the sample (Fig. 5A and fig. S8). PC1 represents 62.2% of the variance and reflects variation in tooth aspect ratio (Fig. 5A). Teeth with low and robust crowns are plotted toward negative PC1, and high, slender teeth are plotted on positive values of PC1. PC2 explains 14.12% of variance, mostly associated with crown curvature and width. Curved and blade-like teeth are plotted on the positive range of PC2, and straight teeth with few cusps are plotted toward the negative range of the axis. PC3 explains 7.52% of variance and reflects differences in dental complexity, separating simple conical teeth toward positive values and complex, multicusped teeth toward negative values. Extant dietary groups show different morphological disparity level as expressed by convex hull volume. Herbivores have the largest convex hull, although not significantly larger than the area of carnivores, while omnivores and insectivores show similar levels of low disparity (figs. S9 and S10). Carnivores occupy a separate and large area of morphospace, mostly located in the positive area of the three PC axes (Fig. 5A), representing curved simple teeth with high aspect ratios, such as the theoretical carnivore shape (Fig. 5B). The large area of morphospace occupied by herbivores is mostly located in the negative area of PC1 (Fig. 5A). The theoretical herbivore tooth shape is lanceolate and multicusped (Fig. 5B). Omnivores largely overlap with herbivores in morphospace, having smaller convex hull area (Fig. 5A) and simple, tricuspid mean shapes (Fig. 5B). Insectivores occupy a central position in morphospace, overlapping the areas of the other three dietary groups (Fig. 5A). Durophages occupy a small area of negative PC1 represented by low bulbous crowns (Fig. 5, A and B). Dinosaurs are mostly restricted to positive PC1 with high aspect ratio teeth, except for *Lesothosaurus*. Early diverging saurischians, such as *Herrerasaurus*, *Eodromaeus*, *Buriolestes*, *Saturnalia*, and *Eoraptor*, are plotted within or close to the carnivore area represented by curved, bladed teeth. Post-Carnian sauropodomorphs occupy an area of morphospace associated with straight, multicusped tooth morphologies with high aspect ratio. Among the ornithischians, *Lesothosaurus* is plotted within the herbivore area of morphospace and *Manidens* occupies a unique area of tall, straight, and multicusped teeth.

The phylogenetic signal in dental morphology is low, as reflected by a *K*_mult_ of 0.5717, but significant (*P* = 0.001). The effect of allometry is not significant in explaining variation in tooth shape when using tooth surface area and volume as size proxies (tables S13 and S14). When centroid size is used, the effect is significant but explains a small proportion of shape variation in the sample (table S12), suggesting a small allometric signal in the shape data. In contrast, diet significantly influences variation in tooth shape within the sample regardless of control for phylogenetic signal, as both standard and phylogenetic Procrustes analysis of variance (ANOVA) recover a significant relationship between diet and shape (*P* = 0.001 and 0.016; tables S15 and S16). Last, dental stress and shape are significantly associated, with and without control for phylogenetic signal (r-PLS = 0.87; *Z* = 4.91; *P* = 0.001).

### Dietary classification

Pairwise permutational multivariate ANOVA (PERMANOVA) was performed on the biomechanical and morphological PC coordinates that account for 90% of the variation to test for significant differences between dietary groups. The results on both datasets reveal that carnivores are significantly different from herbivores, omnivores, and insectivores, but there are no significant differences between the other combinations of groups (table S17). A second PERMANOVA test was carried out with three dietary categories (carnivores, herbivores, and omnivores) by including insectivores and durophages within omnivores based on their overlap in both morphospaces (Figs. 4A and 5A). In this case, all dietary categories were significantly different from each other in both datasets, except for omnivores and herbivores in the morphological dataset (table S18).

**Fig. 6.**
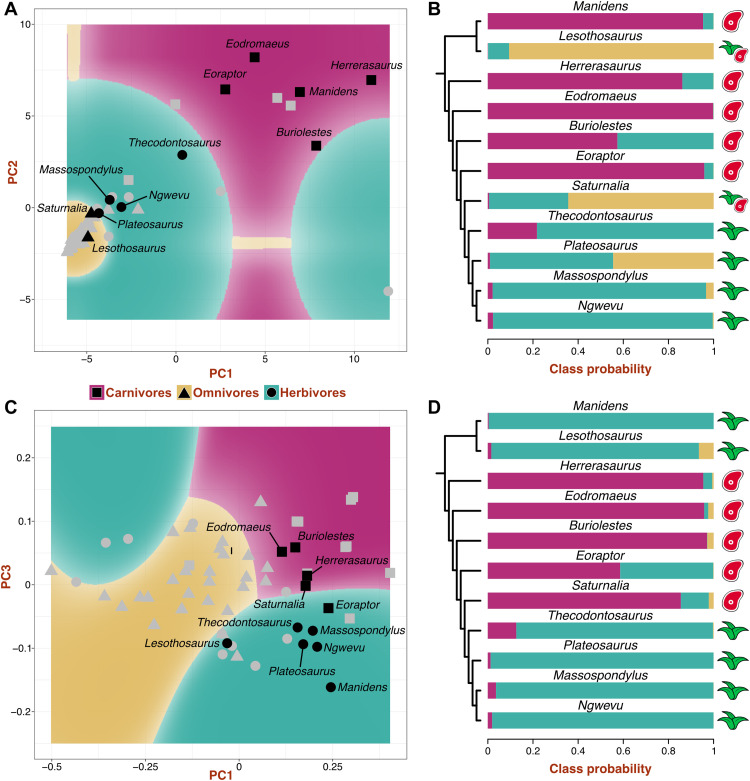
Dietary classification of early dinosaurs. Decision boundary plots from biomechanical (**A**) and morphological (**C**) datasets showing morphospace areas assigned to each dietary category by the best-performing algorithm (naïve Bayes and neural network, respectively). The predictor variables (PCs) with the highest importance in each model are represented. Symbols in gray indicate the dietary category of extant sauropsids, and symbols in black indicate the predicted category of dinosaurs. Dietary class probabilities and predictions for early dinosaurs from biomechanical (**B**) and morphological (**D**) data. Colored bars show class probabilities, and symbols indicate the predicted dietary category.

Nine machine learning algorithms were tested on the biomechanical and morphological datasets, considering three dietary categories. Naïve Bayes was the best-performing one for the biomechanical dataset, and neural network was the best for the morphological dataset (see the Supplementary Materials). After tuning, the biomechanical naïve Bayes model achieved its best performance with the nonparametric method, a Laplace correction of 1, and a bandwidth adjustment of 3 (accuracy, 0.81; Kappa, 0.65). The final neural network model for morphological data achieved its highest accuracy with four hidden layers and weight decay = 0.005 (accuracy, 0.81; Kappa, 0.67). These models were used to generate decision boundary plots of dietary classes over the morphospaces (Fig. 6, A and C) and dietary class probabilities and predictions for the dinosaur sample (Fig. 6, B and D, and table S21). The biomechanical and morphological models agree in classifying *Herrerasaurus*, *Eodromaeus*, *Buriolestes*, and *Eoraptor* as carnivores and *Thecodontosaurus*, *Plateosaurus*, *Massospondylus*, and *Ngwevu* as herbivores (Fig. 6, B and D). *Lesothosaurus* was classified as an omnivore or herbivore; *Manidens* was classified as a carnivore or herbivore, and *Saturnalia* was classified as an omnivore or carnivore.

## DISCUSSION

The morphofunctional analyses of the dentition of extant sauropsids reveal key differences arising from their divergent dietary habits. Extant carnivorous sauropsids have relatively weaker teeth under simple bite forces than species with other diets, as reflected by their higher stress magnitudes and distribution patterns. The lower mechanical resistance to feeding-related forces of carnivorous teeth compared to those of herbivores, for instance, is likely related to the different material properties of meat and plant material (*22*). Unlike flesh, plant material needs to be processed extensively to be digested (*31*), the reason for the generally higher bite forces (*32*) and more intense use of oral food processing (*33*) by herbivores. As the teeth of herbivores are subject to more abrasion, higher tooth replacement rates have evolved in herbivorous dinosaur lineages compared to carnivorous species (*34*). These differences in mechanical properties of food items are also reflected in the theoretical mean tooth shapes of carnivores and herbivores obtained from our sample (Fig. 5B). The generalized carnivore tooth shape is sharp and pointed, suited to puncture and cut ductile and deformable tissues such as vertebrate flesh (*22*, *35*), while the mean herbivorous tooth is blunt and cusped to propagate fractures in tougher materials such as plant tissues (*22*, *31*). Also relevant for dietary inferences in dinosaurs is the overlap of herbivores, omnivores, and insectivores in both the biomechanical and morphological spaces (Figs. 4A and 5A), indicating that the teeth of these groups are similar in shape and stress variation. Herbivores, however, are more diverse in their dental mechanical behavior and shape because of some specialized taxa such as the seaweed-eating *Amblyrhynchus cristatus* (Fig. 4A). These results are in line with studies showing that herbivorous, omnivorous, and insectivorous squamates have teeth that are similar in morphological complexity (*23*, *24*) and microwear patterns (*33*). As noted previously (*23*), these natural similarities hinder the discrimination of these dietary categories, especially separating omnivores from insectivores, among extinct taxa.

Classical inferences of dietary habits in early diverging dinosaurs have been based largely on qualitative comparisons with the dentitions of extant analogs (*10*, *14*, *36*). Our study presents a quantitative analysis of dental morphology and function in early members of the main dinosaurian lineages in comparison with extant analogs, cementing a framework for further dental morphofunctional studies applicable to any fossil sauropsid clade. Quantification of dental shape variation through GMM reveals that the morphological diversity of early dinosaur tooth types spans a wide region of the morphospace occupied by extant sauropsids with different diets and phylogenetic position. Despite the phylogenetic breath of the extant sample, phylogenetic signal in dental shape variation is low. In contrast, tooth morphology is strongly influenced by diet, so that similar dental morphotypes evolved independently in different clades of sauropsids as dietary adaptations. Among dinosaurs, the post-Carnian sauropodomorphs with their denticulated lanceolate teeth (*37*–*40*) are relatively close in morphospace to iguanines, as noted previously from anatomical observations (*10*, *14*), and the early saurischians/theropods and sauropodomorphs are plotted close to extant carnivores with curved, bladed teeth. However, some dinosaurs show unique tooth morphologies, such as *Manidens* with its denticulated “hand-shaped” teeth (*41*). This suggests caution when interpreting diet from 3D dental shape in fossil taxa, even when analyzed quantitatively, due to unexplored dental disparity among extant analogs.

Morphological disparity is linked to variation in biomechanical performance in early dinosaur dentitions, as revealed by stress distribution patterns and magnitudes. Of the dinosaur species studied here, the early saurischians *Herrerasaurus* and *Eodromaeus* and the earliest sauropodomorphs *Buriolestes* and *Eoraptor* have the mechanically weakest teeth (Figs. 2 to 4), indicating that their teeth might have been adapted to process soft food such as animal flesh. Their curved, blade-like teeth resemble morphologically and mechanically those of the extant Komodo dragon *Varanus komodoensis* (Fig. 4A), which are structurally weak against bone crushing, limiting its diet to soft tissues (*42*). While not ideal for crushing, the ziphodont teeth of *Herrerasaurus*, *Eodromaeus*, *Buriolestes*, and *Eoraptor* would have been highly efficient at cutting meat with a “grip-and-rip” action provided by their fine serrations, as demonstrated for other vertebrates with serrated teeth (*43*). In contrast, the early ornithischian *Lesothosaurus*, *Saturnalia*, and plateosaurian sauropodomorphs have more mechanically resistant teeth that may have been adapted to breaking down harder materials such as plant matter or insect exoskeletons (*44*, *45*).

Our machine learning–based classification establishes the first dietary inferences in early dinosaurs based on quantitative morphofunctional data. The early saurischians/theropods *Herrerasaurus* and *Eodromaeus* are consistently classified as carnivores, as well as the early sauropodomorphs *Buriolestes* and *Eoraptor*, all of them sharing curved, blade-like teeth with fine serrations. Herbivory is consistently predicted for the Norian-Rhaetian sauropodomorphs *Thecodontosaurus* and *Plateosaurus* and the Early Jurassic massospondylids *Massospondylus* and *Ngwevu*. These results agree with dietary inferences on the basis of the morphology of their denticulated, lanceolate teeth, which favor omnivory or herbivory (*10*, *14*, *46*, *47*). Among ornithischians, *Lesothosaurus* is predicted to have been either an omnivore or an herbivore. *Manidens* is unexpectedly recovered as a carnivore by the biomechanical model. This is likely the result of analyzing isolated teeth in a species that had evolved a dental battery composed of closely packed, high crowned teeth as an adaptation to herbivory (*48*, *49*). Because of its unique dental morphology, the isolated teeth of *Manidens* experience high stresses, as in extant carnivores.

The origin and ascent of dinosaurs during the Triassic and Early Jurassic are of longstanding interest in macroevolution (*50*). The current noncompetitive model of early dinosaur evolution (*51*–*53*) encompasses the fact that certain dinosaurian traits, such as increased growth rates, contributed to the differential survival and success of this clade (*53*, *54*). The evolution of feeding adaptations in these early stages has been discussed before, although their role in the ecological diversification and evolutionary radiation of Dinosauria remains unclear (*2*, *10*, *11*, *55*). Our study is the first to recover quantitative morphofunctional evidence for the notable dietary diversity in the earliest dinosaurs: Early saurischians/theropods are classified as carnivores; sauropodomorphs evolved diverse feeding habits from ancestral carnivory, and omnivory might have been the dietary condition of the earliest ornithischians. These findings suggest that obligate herbivory was acquired late in the evolution of sauropodomorphs and ornithischians and was not associated with the early divergence of dinosaur clades. We propose that this diversity of diets might have been a key contributor to the evolutionary success of dinosaurs through the Late Triassic and Early Jurassic.

The evolution of feeding modes is particularly complex in Sauropodomorpha. Early members of this clade were traditionally considered herbivores based on their dental similarity with iguanine squamates, as well as other cranial and postcranial features (*14*). The current view, however, is that strict herbivory evolved in later diverging taxa, associated with the acquisition of quadrupedality and giant body size, while early sauropodomorphs retained omnivorous habits (*10*, *46*, *47*). Recent discoveries of a plethora of Carnian sauropodomorphs in South America support this hypothesis, as they exhibit wide variation in dental morphologies (*11*, *17*, *20*). In particular, the strongly “carnivorous-like” tooth morphology of the earliest diverging taxon, *Buriolestes*, was proposed as evidence for ancestral carnivory in dinosaurs (*11*). Our analyses of dental shape and mechanical resistance find the first quantitative evidence for carnivory in the Carnian species *Buriolestes* and *Eoraptor*. This agrees with previous dietary interpretations of *Buriolestes* (*11*, *56*), although the diet of *Eoraptor* was thought to have been more ambiguous on the basis of their slightly less curved and basally constricted tooth crowns (*19*, *20*). The Carnian *Saturnalia* shows intermediate dental morphofunctional traits. On the basis of its craniodental morphology and neuroanatomy, *Saturnalia* had been proposed as a predator on insects or small vertebrates (*20*, *57*), in agreement with the present predictions of faunivory or omnivory in this taxon. Our analyses recover functional similarity between post-Carnian sauropodomorphs and extant herbivores, providing quantitative evidence for the idea that this clade evolved morphofunctional dental traits consistent with herbivory by the latest Triassic. The Rhaetian species *Thecodontosaurus* has been interpreted as an herbivore with occasional faunivorous habits based on its osteology and neuroanatomy (*40*, *58*). The present predicted herbivory for *Thecodontosaurus* suggests that, regardless of the possible retention of predatory habits, its dentition was well suited to process plant matter. Plateosaurians are also classified as herbivores, although the dental mechanical performance of *Plateosaurus* suggests some similarities with omnivores (high probability of omnivory; Fig. 6B and table S21) that might reflect occasional consumption of animal matter. In contrast, later sauropodomorphs such as the massospondylids have dentitions better suited for herbivory. Non-sauropodan sauropodomorphs have been interpreted as unspecialized herbivores that retained occasional faunivorous habits after acquiring “herbivorous” adaptations such as coarsely denticulated, lanceolate teeth (*2*, *10*, *46*, *47*). Thus, obligate herbivory evolved in later diverging taxa close to Sauropoda in association with notable increase in body size and a shift to a quadrupedal stance (*46*). Our results indicate that by the Norian, sauropodomorphs had already acquired dental adaptations to an herbivorous diet, particularly Early Jurassic taxa, and thus, occasional faunivory in species such as *Thecodontosaurus* and *Plateosaurus* might have been facilitated by behavior rather than craniodental adaptations (*58*).

The origin of ornithischians has long been linked to herbivory from the start (*16*), and many ornithischian synapomorphies are related to the craniodental apparatus (*59*, *60*). Against this traditional view, functional interpretations of one of the earliest ornithischians, *Lesothosaurus*, suggested that its tooth morphology and weak dental wear (*36*, *61*) indicated omnivorous habits, which might be the most likely plesiomorphic condition of Ornithischia (*10*, *62*). Our analyses suggest that *Lesothosaurus* had a predominantly herbivorous diet, likely composed of soft plant matter (*63*), with occasional omnivorous habits. Despite such omnivory in the earliest ornithischians, a highly efficient craniodental apparatus, including dental batteries, evolved in Early Jurassic clades such as heterodontosaurids (*55*, *64*), indicating a shift to herbivory in some early ornithischian lineages. Among heterodontosaurids, *Manidens* has been described as having intermediate craniodental traits (*41*, *48*), with an incipient dental battery compared to *Heterodontosaurus* (*64*) but with an efficient jaw apparatus to process plant material (*65*). Our prediction based on tooth morphology is in line with this evidence, although *Manidens* is here classified as a carnivore based on tooth mechanics. While we think this emerges from analyzing isolated teeth, it must be noted that occasional faunivory has been proposed for heterodontosaurids based on their enlarged caniniform teeth (*66*). Nonetheless, the complex jaw apparatus and mechanics of heterodontosaurids (*64*, *65*), and even the tooth occlusion of *Lesothosaurus* (*55*, *61*), indicate that the earliest ornithischians were more efficient at plant processing than most non-sauropodan sauropodomorphs. This, however, did not translate into a greater evolutionary success of ornithischians; on the contrary, sauropodomorphs were more diverse and abundant during the Late Triassic and Early Jurassic (*2*).

The scenario of dietary evolution in ornithischians is dependent on the phylogenetic position of silesaurids. This clade is usually placed as the sister clade of Dinosauria (*8*), but it could represent a paraphyletic group at the base of Ornithischia (*9*, *11*). While the earliest diverging silesaurid has a carnivorous-like dentition, later species have been interpreted as herbivores on the basis of their craniodental similarities with ornithischians (*9*). However, evidence from coprolites suggests that some of these putative herbivores were likely insectivorous (*21*). Thus, if silesaurids are a paraphyletic assemblage within Ornithischia, then the early diversity of feeding modes and dietary evolution in this clade would become much more complex than previously thought; from carnivorous origins, most “silesaurids” and early ornithischians became omnivorous, and some later lineages evolved predominantly herbivorous habits.

Dietary evolution has been central in models of early dinosaur evolution despite the intricate scenario (*67*). Traditional hypotheses proposed that dinosaurs originated from carnivorous ancestors, and theropods retained this habit while key adaptations for herbivory were already present in Triassic sauropodomorphs and ornithischians (*16*, *59*). However, other authors consider that omnivory was widespread among early dinosaurs and was an equally possible ancestral condition for Dinosauria (*1*, *10*, *46*, *62*). Our study provides previously unknown information to infer the ancestral dinosaur diet, although this is contingent on the phylogeny of early dinosaurs. If *Herrerasaurus* and *Eodromaeus*, here predicted to be carnivores, are early members of Saurischia (*8*, *11*) and Ornithischia is its sister group, then the ancestral diet of dinosaurs could either be carnivory or omnivory. Conversely, under the Ornithoscelida hypothesis, where Ornithischia and Theropoda are sister groups, the carnivorous *Eoraptor* and *Eodromaeus* are the earliest branching theropods, and herrerasaurids are the sister group of Sauropodomorpha (*7*). In this scenario, the distribution of dietary preferences predicted in our analyses suggests that carnivory is the most likely ancestral diet of Dinosauria. Thus, resolving the phylogenetic relationships at the base of Dinosauria is a fundamental requisite to reconstruct the pattern of dietary evolution. Future studies should assess the polarity of dental morphofunctional trait changes and the evolution of dietary habits at the base of Dinosauria with phylogenetic methods of character reconstruction.

Our analyses provide quantitative evidence for previously unrecognized functional diversity in the dentitions of early dinosaurs, akin to modern sauropsids with different diets. Sauropodomorpha underwent a dietary shift, with its earliest members showing dental characteristics associated with an exclusively or predominantly faunivorous diet, some Carnian species experimenting with diverse diets, and post-Carnian lineages acquiring dental adaptations toward herbivory. The early diverging ornithischian *Lesothosaurus* is reconstructed as an herbivore with occasional omnivorous habits, further supporting the hypothesis of a late shift to obligate herbivory in Ornithischia. The varied dental adaptations acquired by members of different dinosaurian lineages and their conquest of diverse dietary niches during the Late Triassic and Early Jurassic contributed to the later adaptive radiation of Dinosauria.

## MATERIALS AND METHODS

### 3D modeling

The tooth 3D model dataset comprised 47 extant sauropsids (44 squamates and 3 crocodilians) and 11 early dinosaurs (Fig. 1). Models were created from existing datasets (table S1) downloaded from MorphoSource (www.morphosource.org). Details of the early dinosaur dataset can be found in the Supplementary Materials. Teeth were either isolated from mandible surface models using Blender 2.92 (Blender Foundation) or segmented from computed tomography (CT) data in Avizo Lite 9.5 (Thermo Fisher Scientific). Tooth selection within mandibles was based on preservation and tooth row position criteria, so models capture the characteristic dental features of each taxon. Thus, the best-preserved teeth from the middle to posterior part of the right dentary were chosen. When these were not available, posterior teeth from the left dentary or the maxillae were mirrored to resemble a right dentary tooth. Tooth models were prepared in Blender, and models were reoriented consistently along the mesiodistal axis of the mandible. The crowns were isolated, and roots were removed at the level of the mandible using a Boolean modifier. The tooth surfaces were smoothed using the Smooth and Draw tools in the Sculpting panel. After cleaning, tooth models were scaled up to the same surface area (1000 mm^2^). Surfaces were remeshed using the Remesh modifier, selecting a uniform voxel size value of 0.15 mm, decided from the convergence test required for FEA (see below and also the Supplementary Materials). The Triangulate modifier was applied to the resulting meshes to transform quads into triangles. Mesh quality checks were run in Edit mode to identify and remove mesh artifacts. The final 2D meshes had the same surface area and a similar number of triangles (table S2).

Our sample includes species with a wide range of body and tooth sizes. Absolute tooth size is an important factor in tooth performance at processing food (*44*), and it should not be omitted when inferring detailed aspects of tooth mechanics. However, standardization to the same scale is common in comparative analyses that include taxa ranging greatly in body size (*23*, *26*, *45*). Scaling is also a requirement of our analyses, as GMM scales landmark coordinates during the Procrustes alignment, and meaningful comparisons of fine element (FE) models require a constant force–to–surface area ratio. Thus, we measured the original size of tooth models before scaling (tooth surface area and volume; table S2) to be included in downstream analyses to account for the influence of allometry in our results. Extant sauropsids and dinosaurs are not significantly different in tooth size, as reflected by a two-sample Student’s *t* test on tooth surface area (*t* = 1.66, *P* = 0.1) and volume (*t* = 0.97, *P* = 0.34).

### Finite element analysis

Tooth surface models were imported into HyperMesh 2017 (Altair) and tetrameshed, producing 3D meshes composed of a similar number of C3D4 tetrahedral elements (table S2). The appropriate number of elements in the 3D mesh was determined in a convergence test to minimize errors across models due to mesh resolution (see the Supplementary Materials). Meshes were assigned material properties of bovine dentine (*68*) and were considered elastic, homogeneous, and isotropic for comparative purposes. Boundary conditions and loads were applied in Abaqus 6.14 (Simulia). Twenty nodes equally spaced around the external margin of the tooth base were constrained in all degrees of freedom in a multipoint constraint, with a reference point located below the center of the base. The number of constrained nodes around the base and the position of the reference point did not influence the results (see the Supplementary Materials). The ratio between applied force and model surface area was kept constant across models to compare the relative biomechanical performance of the different tooth shapes (*69*). A vertical and basally directed load of 100 N was applied to one node at the tip of the tooth crown, simulating a simple bite force. An exception was made in the *Dicrodon guttulatum* FE model, in which a total load of 100 N was divided between two 50-N loads applied to one node on each of the two main cusps of the tooth.

Von Mises stress and element volume are reported for each element in the mesh as output parameters of the analysis. Von Mises stress is commonly used in biomechanics because it reflects the tendency of a material to undergo ductile failure (*69*, *70*). The average von Mises stress of each model was calculated using the MWAM, which accounts for mesh heterogeneity and artifactual high stress values by considering element volume (*30*). MWAM calculations were run in R (*71*), modifying a published code (*72*). It must be noted that these analyses are not designed to predict absolute stress magnitudes accurately but to compare relative patterns of stress distribution and magnitudes across different tooth shapes.

Multivariate statistics were applied to the stress data using the intervals method, which divides the elements into stress intervals and analyzes these newly generated variables in a PCA (*73*). The minimum number of intervals required to reach convergence was 150 (fig. S3). The correlation matrix of the intervals data was submitted to a PCA to summarize and visualize the biomechanical data in a biomechanical space. All steps of the intervals method were run in R (*71*) using the protocol and code from the original publication (*73*). Biomechanical disparity was measured as convex hull volume per dietary category (except for durophages due to their small sample size) using the dispRity R package (*74*), and differences between groups were assessed through pairwise comparisons using permutation tests with 1000 iterations (figs. S5 and S6).

### Geometric morphometrics

Tooth 3D shape was analyzed using landmark-based GMM. Fixed landmarks and curve semilandmarks were digitized in IDAV Landmark Editor 3.0 (*75*). Three fixed landmarks were placed on the tip of the crown and the mesial and distal-most points on the base perimeter. Two curves delimited the crown base perimeter, and two curves were placed along the mesiodistal carinae. Curve semilandmarks were resampled for even spacing (*76*). Surface semilandmarks were digitized on the labial and lingual surfaces of a template model in Landmark Editor. The template was modeled in Blender to resemble a hypothetical simple tooth shape (fig. S7). Surface semilandmarks were transferred from the template to the tooth models using the patching procedure in the placePatch function of the Morpho package in R (*77*). Curve and surface semilandmarks were slid to optimize the correspondence of landmark positions (*78*) using the relaxLM and slider3d functions in the Morpho package (*77*). Four sliding steps were performed, with the first step minimizing the bending energy of a thin plate spline between each specimen and the template and the last three steps minimizing the bending energy of a thin plate spline between the result of the first sliding and the Procrustes consensus of the sample (*79*). These four sliding steps were performed to make semilandmarks geometrically homologous (*79*), and bending energy was chosen because it performs better when working with large shape variation (*78*). Three fixed landmarks, 44 curve semilandmarks, and 98 surface semilandmarks were used in the analyses (Fig. 5C and fig. S7).

The final landmark coordinates of all specimens were aligned in a generalized Procrustes analysis (GPA) to remove the effects of size and position by scaling, translating, and rotating the landmark configurations. The resulting Procrustes coordinates were ordinated in a PCA to summarize and visualize shape variation. Both GPA and PCA were performed using the R package geomorph (*80*). The extreme tooth shapes of PC1 to PC3 were recreated by warping the model of the specimen closest to the mean of the Procrustes alignment (*Hemitheconyx caudicinctus*) with the Procrustes coordinates of the extremes using the warpRefMesh function in geomorph. We also reconstructed the hypothetical mean tooth shapes of each dietary category with the same approach using the mean of the Procrustes coordinates of each dietary category from the mshape function. The morphological disparity of each dietary group (except for durophages) was computed as convex hull volume using dispRity (*74*), and pairwise comparisons between groups were assessed through permutation tests with 1000 iterations (figs. S9 and S10).

### Statistical analyses

The importance of phylogenetic signal in tooth stress variation was tested using *K*_mult_, the extension of Blomberg’s *K* statistic for multivariate data (*81*), on both MWAM and stress intervals. For these tests, we used a time-calibrated informal supertree of Sauropsida (fig. S11, table S5, and the Supplementary Materials). We tested the effect that allometry has on structuring dental stress variation with phylogenetic generalized least squares (PGLS) regressions of stress (MWAM and intervals) on tooth size (log centroid size, log surface area, and log tooth volume) for multivariate data (*82*).

The effect of phylogeny, tooth size, and diet on variation in tooth morphology was assessed with different statistical tests on the GMM results. We tested for phylogenetic signal in shape variation, indicated by *K*_mult_ (*81*), applying the physignal function in geomorph to the Procrustes coordinates. The importance of allometry was tested using a PGLS of Procrustes coordinates on tooth size (log centroid size, log surface area, and log tooth volume) using the procD.pgls function in geomorph. We also tested the influence of diet in dental shape variation with standard and phylogenetic Procrustes ANOVA tests on the Procrustes coordinates using the geomorph functions procD.lm and procD.pgls, respectively. The association between dental shape and stress was tested with two-block PLS and PGLS regressions of Procrustes coordinates and stress intervals with 1000 iterations using the two.b.pls and phylo.integration functions in geomorph (*80*).

Differences in dental morphology and stress among dietary categories were tested in PAST 4.09 (*83*). Two pairwise PERMANOVA tests with Bonferroni correction for multiple comparisons were performed on the biomechanical and morphometric results using Euclidean distances. The test was performed on the PC scores that accounted for 90% of the variance; the first two PCs are from the biomechanical analysis, and the first five PCs are from the morphometric analysis. The tests were performed by considering the five and three dietary categories and the latter by combining omnivores, insectivores, and durophages.

### Machine learning classification

The diet of early dinosaurs was predicted by applying machine learning algorithms to the biomechanical and morphological data, following the workflow proposed in (*28*, *29*). The classification was conducted on the PC coordinates corresponding to the PCs that accounted for 90% of the variance (two PCs for biomechanical data and five PCs for morphological data) and the dietary information from living species, divided into three classes (carnivores, herbivores, and omnivores). The extant dataset was split into training and testing datasets, the former representing 75% of the dataset.

Machine learning classification was performed using the caret package in R (*84*). Nine algorithms were tested initially using a leave-group-out cross-validation with 200 repeats and an automatic grid search to tune the algorithm parameters. Because of the imbalanced numbers of each class within the classification sample (10 carnivores, 27 omnivores, and 10 herbivores), we repeated the tests using up-sampling during cross-validation (*85*) and assessed its impact on the results. The model performance was assessed using accuracy and Kohen’s Kappa (fig. S12 and tables S19 and S20) (*85*). The best-performing algorithm in the biomechanical dataset was neural network, followed by naïve Bayes (table S19), although the performance of both models was not significantly different (*P* = 1 for accuracy and Kappa). Naïve Bayes was chosen for the classification tasks because of its higher balanced accuracies for the minority classes (1 for carnivores and 0.875 for herbivores) compared to neural network (0.938 and 0.438, respectively). Up-sampling had a nonsignificant effect in biomechanical models; for instance, it did not significantly improve the accuracy (0.73 to 0.74; *P* = 0.002) and Kappa (0.49 to 0.53; *P* = 1.1 × 10^−5^) of the naïve Bayes model. Of the morphological classification models, neural network is the best performing (table S20) and has significantly different accuracy and Kappa values from the rest of the models except for random forest (*P* = 1 for accuracy and *P* = 0.35 for Kappa). Neural network was chosen for classification because of its overall higher performance and higher balanced accuracy values for the minority classes (1 for carnivores and 0.5 for herbivores) compared to random forest (0.75 for carnivores and 0.625 for herbivores). Up-sampling had a nonsignificant effect in the accuracy of the morphological neural network model (0.74 to 0.71; *P* = 6 × 10^−4^) but significantly improved its Kappa (0.49 to 0.5; *P* = 0.6).

The best-performing algorithm for each dataset (naïve Bayes for biomechanical data and neural network for morphological data) was manually tuned with a grid search to increase its classification accuracy using up-sampling and leave-one-out cross-validation (*85*). Grid search is an exhaustive and widely used method to test all combinations of set ranges of values of model parameters, feasible for models with few parameters and easily incorporated in caret (*84*). During tuning, the range of weight decay values was set from 0 to 0.1 to penalize overfitting of the data in a complex model such as neural network. The final mean accuracy and Kohen’s Kappa for the biomechanical model are 0.81 and 0.65, respectively, and the predictor variables with the most importance are PC1 and PC2. The morphological model has a mean accuracy and kappa of 0.81 and 0.67, respectively, with PC3 and PC1 being the variables with the most importance. The models achieved balanced accuracies of 1 for carnivores, 0.875 for herbivores, and 0.833 for omnivores. The final models were used to predict the class probabilities and class predictions of the dinosaur sample. Because low but significant phylogenetic signal is present in both biomechanical and morphological datasets, the classification models may be affected by the phylogenetic structure of the data. Decision boundary plots were created using the two variables with the highest importance in each model to establish class predictions in 2D morphospaces, following Püschel *et al.* (*28*, *29*).

## Supplementary Materials

### This PDF file includes:

Supplementary Text Figs. S1 to S12 Tables S1 to S21 References

## Notes

View/request a protocol for this paper from *Bio-protocol*.
