# Supplementary material for: Dental form and function in the early feeding diversification of dinosaurs
Source: Sci Adv. 2022 Dec 16;8(50):eabq5201. doi: 10.1126/sciadv.abq5201 (PMC9757754; doi:10.1126/sciadv.abq5201)
Supplement: Supplementary file 1 — Supplementary Text Figs. S1 to S12 Tables S1 to S21 References [file sciadv.abq5201_sm.pdf]

Science Advances
AAAS

Supplementary Materials for

Dental form and function in the early feeding diversification of dinosaurs

Antonio Ballell, Michael J. Benton, Emily J. Rayfield

Corresponding author: Antonio Ballell, antonio.ballell@bristol.ac.uk

Sci. Adv. **8**, eabq5201 (2022)
DOI: 10.1126/sciadv.abq5201

This PDF file includes:

Supplementary Text
Figs. S1 to S12
Tables S1 to S21
References

Supplementary Text

Dinosaur tooth 3D modelling

Eleven early branching members of Dinosauria were included in this study. Maxillary or dentary teeth were selected to build the models based on preservation. Premaxillary teeth were omitted as their morphology differs from that of the cheek dentition in most taxa. Models were created from skull CT data segmented in Avizo Lite 9.5 (Thermo Fisher Scientific) and tooth surface models were cleaned and edited in Blender 2.92 (Blender Foundation) to recreate dental morphology guided by relevant literature. None of the dinosaur species studied show notable dental wear, except for *Manidens* and, to a lesser extent, *Lesothosaurus*.

Manidens condorensis is a heterodontosaurid ornithischian from the Early Jurassic of South America (48, 49). The dentition of *Manidens* shows marked heterodonty, with diamond-shaped maxillary teeth and closely packed hand-shaped dentary teeth, as well as a pair of anterior dentary caniniform teeth (41, 48, 65). Our reconstruction of *Manidens* tooth shape is based on CT scans of the holotype MPEF-PV 3211 (49), from which maniform dentary teeth were selected due to their autapomorphic and complex morphology. In particular, the right ninth dentary tooth was segmented, which lacks any wear facets (49), and later repaired based on the rest of the dentary dentition from the CT data as well as images and descriptions of *Manidens* dentary teeth (41, 48).

Lesothosaurus diagnosticus is an early branching ornithischian from the Early Jurassic of southern Africa (15, 87, 88). The teeth of *Lesothosaurus* have triangular crowns with multiple cusps along the mesiodistal margin. The crown is constricted at the base and shows the characteristic cingulum present in many ornithischians. *Lesothosaurus* does not show evident heterodonty along the tooth row (61). The tooth model corresponding to this species was obtained from the left dentary of specimen BP/1/7853 (61). The fourteenth dentary tooth was segmented for being the best preserved and lacking any notable wear (61). Minimal repair was guided by other teeth in BP/1/7853 and other specimens (15, 61).

Eodromaeus murphi is an early branching saurischian or theropod from the Carnian (Late Triassic) of South America (8, 13). *Eodromaeus* is characterized by having curved and laterally compressed teeth which are finely serrated on their mesial and distal carinae. Dental morphology does not vary along the toothrow, although some maxillary teeth are enlarged into caniniforms (13). The *Eodromaeus* tooth model was obtained from CT scans of the holotype PVSJ 560, represented by disarticulated skull material. The best-preserved dentary tooth, belonging to the right mandible, was segmented and the resulting surface file was cleaned and repaired based on descriptions and images of the dentition (13).

Herrerasaurus ischigualastensis is a herrerasaurid dinosaur from the Carnian (Late Triassic) of South America (12, 89). Its teeth are laterally compressed and curved, with a rounded mesial surface on the basal half of the crown and a sharp distal carina. Fine serrations are present all along the distal carina and along the apical part of the mesial carina. Dental morphology is relatively uniform along the toothrow, although size variation is notable among the maxillary dentition (12). A CT dataset of the specimen PVSJ 407 was the basis of our *Herrerasaurus* tooth reconstruction. The eleventh right dentary tooth was segmented due to its better preservation and later edited to remove resolution artefacts and add morphological details described in the original study of the species (12).

Buriolestes shultzi is the earliest branching sauropodomorph from the Carnian (Late Triassic) of South America (11, 90). The dentition of *Buriolestes* is characterized by the presence of curved, laterally compressed teeth bearing fine serrations along the distal and mesial carinae. Both maxillary and dentary toothrows show size heterodonty (11, 90). The *Buriolestes* tooth model was created from CT data of CAPPA/UFSM 0035 (56), selecting a middle right maxillary tooth for its preservation. The segmented surface file was cleaned and mirrored to resemble a right dentary tooth. It was also modified to reflect the characteristic set of traits of the species based on descriptions and photographs (11, 90).

Eoraptor lunensis is an early dinosaur from the Carnian (Late Triassic) of South America (19), usually recovered as one of the earliest branching sauropodomorphs (8, 11, 90, 91) but occasionally classified as an early theropod (7). The dentition of *Eoraptor* is composed of laterally compressed tooth crowns that show a basal constriction and are only slightly recurved. Tooth crowns show a labial rounded eminence and serrations along the distal carina and on the apical half of the mesial carina (19). CT scans of the skull of the holotype PVSJ 512 were segmented to isolate a representative tooth model, choosing the well-preserved sixth left maxillary tooth. The model was edited to include fine details like the serrations, which are not captured in the CT scans, following photographs and descriptions of the specimen (19).

Saturnalia tupiniquim is an early member of Sauropodomorpha from the Carnian (Late Triassic) of South America (92). Several aspects of the dentition of *Saturnalia* are unknown due to the generally poor preservation of the cranial material. However, well-preserved maxillary and dentary crowns show a basal constriction followed by a mesiodistal expansion of the crown, which tapers apically. The mesial margin is anteriorly convex while the distal margin is straight to sigmoid, producing a slight posterior curvature of the teeth. The distal margins of the best-preserved teeth show fine denticles (20). The *Saturnalia* tooth model was obtained from CT data of the specimen MCP-3845-PV, selecting a well-preserved right dentary tooth, approximately the ninth along the tooth row. The surface model was cleaned, and details missed from the CT scans were modelled according to published descriptions and images (20, 57).

Thecodontosaurus antiquus is an early branching sauropodomorph from the Rhaetian (Late Triassic) of Europe (40, 93). The dentition of *Thecodontosaurus* is composed of lanceolate crowns with coarse denticles on the mesial and distal margins. The maxillary and dentary crowns are slightly posteriorly inclined and show a basal constriction and a tapering apex. Both the labial and lingual surfaces bear a central apicobasal eminence which is more developed on the labial side (40, 93). The *Thecodontosaurus* tooth model was created from CT scans of the specimen BRSUG 28221, a fragmentary left maxilla (40). A posterior left maxillary tooth was segmented and later cleaned to remove segmentation artefacts.

Plateosaurus engelhardti is an early sauropodomorph dinosaur from the Norian (Late Triassic) of Europe (38). *Plateosaurus* maxillary and dentary teeth are lanceolate, with convex mesial and distal margins in labial view. These margins bear coarse and prominent denticles along their length, as well as a labial eminence along the apicobasal axis of the crown. Tooth crowns are almost straight and constricted at their base (38). The source of the *Plateosaurus* tooth model is CT data of the specimen MB.R.1937, a complete skull (47), from which the fourteenth left dentary tooth was isolated. The surface modelled was mirrored to the right counterpart and was cleaned and smoothed to remove resolution artefacts. Fine dental details were modelled based on photographs, drawings and descriptions of *P. engelhardti* specimens (14, 38) and other *Plateosaurus* species (94, 95).

Massospondylus carinatus is a massospondylid sauropodomorph from the Early Jurassic of southern Africa (37). The cheek teeth of *Massospondylus* are lanceolate, apicobasally elongate and constricted at the base. Crowns bear apically oriented denticles on the apical part of the mesial and distal carinae. Variation in size is seen along the tooth row as crown decrease in height towards the posterior end (37, 96). Our *Massospondylus* tooth reconstruction is based on CT data of the specimen BP/1/5241, a complete cranium (96). The eighth left maxillary tooth was segmented due to its preservation. The surface model was smoothed and the denticles, not captured in the scans, were modelled based on available information of *Massospondylus* specimens (37, 96).

Ngwevu intloko is a massospondylid sauropodomorph from the Early Jurassic of southern Africa (39, 96). Dental morphology in *Ngwevu* is similar to that of *Massospondylus* in having maxillary and dentary teeth with lanceolate and elongated crowns that are constricted at their base. Coarse denticles are present on the apical part of the crowns. The *Ngwevu* tooth model was obtained from CT scans of the skull of the holotype BP/1/4779. The eleventh right maxillary tooth was segmented and later cleaned, based on photographs of the specimen (39), and mirrored to resemble a right dentary crown.

Constraint test

A test was performed to assess the impact of varying the number of constraint nodes and the position of the reference point in the multi-point constraint at the base of the crown. The *Iguana iguana* tooth model was tetrameshed in Hypermesh 2017 (Altair), resulting in a 3D mesh composed of 3296634 C3D4 tetrahedral elements. Material properties of bovine dentine – Young's modulus of 21 GPa and a 0.31 Poisson's ratio (68) – were assigned to the model. Boundary conditions and loads were applied were applied in Abaqus 6.14 (Simulia). We used a multi-point constraint to 'fix' the base of the tooth crown during the simulation. This involves a defined number of constraint nodes around the tooth base connected to a fixed reference point node. The test looked at the effect on tooth stress distribution and magnitude of using different numbers of nodes to constrain movement at the base of the tooth crown. A vertical and basally-oriented force of 100 N was applied to a node at the tip of the tooth crown. Ten simulations were performed, varying the number of constraint nodes: 20, 40, 60, 80 and all (378) nodes – and the position of the reference point along the apicobasal (y) axis from either immediately below the crown base surface to a distance equal to the crown height, representing the root. The multi-point constraint was constrained in all degrees of freedom, both in translation and rotation. Von Mises stress and element volume were obtained from the analyses, and these were used to calculate the mesh-weighted arithmetic mean Von Mises stress of models (30).

The results of the constraint test confirm that varying the position of the constraint reference point has no effect on stress values, and variations in this magnitude resulting from increasing the number of constraint nodes is not statistically significant, with error percentages below 1% (fig. S1, table S3). This demonstrates that the number of slave nodes and the position of the reference point in the multi-point constraint has no significant impact on stress magnitude and distribution in our FE models.

Convergence test

A convergence test was carried out to decide the element size and approximate number of elements of the mesh that should be used in the simulations, in order to minimize errors due to mesh resolution across models. The *Iguana iguana* tooth model was used in the test and the 2D

mesh was remeshed in Blender 2.92 with varying edge lengths to generate seven surface models with different resolution (table S4). These surface models were tetrameshed in Hypermesh 2017, generating meshes composed of different number of C3D4 elements (table S4), and material properties of bovine dentine were assigned to them (68). Boundary conditions and loads were applied on Abaqus 6.14, using a multipoint constraint composed of 20 nodes located around the exterior edge of the crown base and a reference point placed right below the centre of the base. The base was constrained in all degrees of freedom and a vertical, basally oriented force of 100 N was applied on a node at the tip of the crown. Von Mises stress and element volume was requested as analysis output parameters to calculate the mesh-weighted arithmetic mean of Von Mises stress (30).

Meshed ranging from approximately 10 million to 600,000 elements, generated with element edge lengths from 0.05 to 0.2 mm, yield similar von Mises stress values (errors below 3%, fig. S2, table S4), indicating that convergence is achieved within this range of mesh resolution.

Phylogeny time-calibration

We built an informal supertree combining the phylogenetic topologies of extant squamates (97), crocodylians (98) and non-avian dinosaurs (9) (fig. S11). We time calibrated the phylogeny using occurrence data from the Paleobiology Database (fossilworks.org) and original sources (13, 99, 100), constraining the calibration with minimum node ages of eight clades (98, 101, 102) (table S5). Time calibration was performed using the timePaleoPhy function of the paleotree package (103) in R.

Machine learning algorithms information

The nine machine learning algorithms used in this study are briefly described below based on Kuhn and Johnson (85).

Classification and Regression Trees (CART) partition the data into smaller groups and fit a simple model to each of them (104). These subgroups are more homogeneous in the response variable. CART are graphically represented with decision trees. The parameters that can be tuned are the complexity of the tree (cost complexity), the minimum number of splits and the maximum depth of the tree.

Linear Discriminant Analysis (LDA) finds the linear combination of predictor variables that maximizes between-group variance in relation to within-group variance (105, 106). Thus, LDA looks for the combination of predictors that maximizes the separation between the centre of the groups and minimizes the variation within each group. LDA does not require tuning.

Mixture Discriminant Analysis (MDA) is an extension of LDA that allows a set number of subclasses within each class (107). The tuning parameter is the number of subclasses.

Support Vector Machines (SVM) separate classes of the dependent variable by defining boundaries between groups and maximizing the margin (i.e., the distance between the boundary and the closest observation within the training sample) (108). Boundaries are drawn using kernel functions which can be linear, polynomial or radial. The main tuning parameter is the cost, which is the penalty for misclassification caused by reducing the margin. Here, we use linear (SVML) and radial (SVMR) support vector machines.

k-Nearest Neighbor (kNN) establishes classifications based on the distance of a sample to the neighbors of each class (109). The sample is classified in the group that is more frequent

among the neighbors within a given distance. The tuning parameter for this algorithm is the number of neighbors (k).

Naïve Bayes (NB) applies Bayes' probability rule to calculate the probability of belonging to a class given the predictor variables in the dataset (110). The model assumes that the predictor variables are independent. The tuning parameters for NB deal with the use of kernel density estimates and Laplace correction, that prevents predictors without train set samples to make the posterior probability zero.

Random Forest (RF) creates a series of classification trees that are obtained from bootstrap resamples of the data, making the trees independent and decorrelated. The class probability of a sample is derived from the distribution of individual classifications made by each tree in the forest (111). The tuning parameter is the number of randomly selected predictor variables to choose from at each split (mtry).

Neural Networks (NNet) combine predictor variables into a set of hidden units that conform one or more hidden layers (112). Information from the hidden layer(s) is combined to produce the classification output. A cost function is used to assess classification error and improve the model via back propagation. The main tuning parameters are the number of hidden units (size) and the weight decay (decay), which penalizes overfitting.

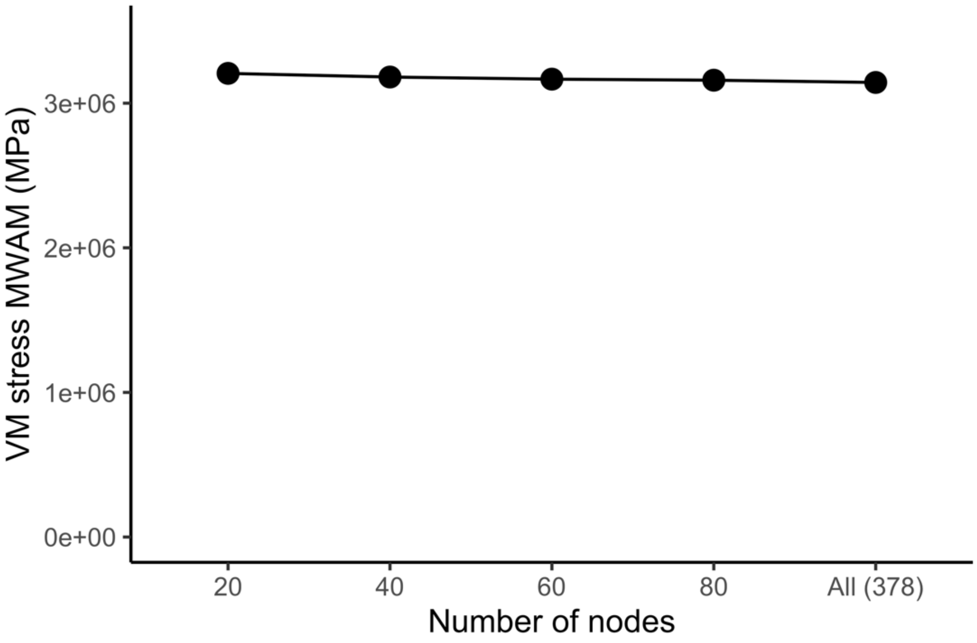

Fig. S1. Constraint test. Variation in von Mises stress mesh-weighted arithmetic mean (MPa) with varying number of constrained nodes around the base of the tooth FE model of *Iguana iguana*.

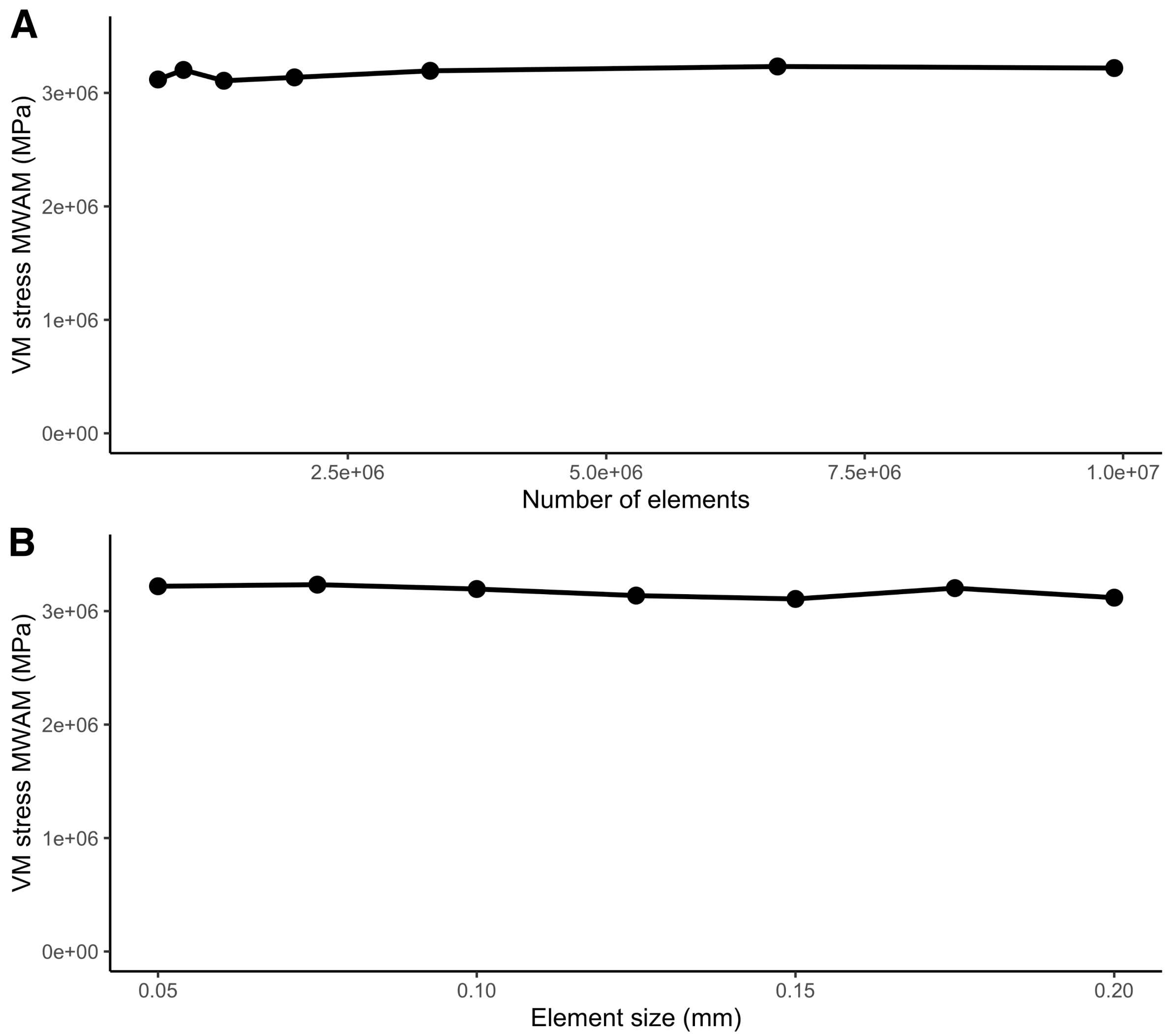

Fig. S2. Convergence test. Variation in von Mises stress mesh-weighted arithmetic mean (MPa) with varying a number of tetrahedral elements and b corresponding mean element size in mm in the tooth FE model of *Iguana iguana*.

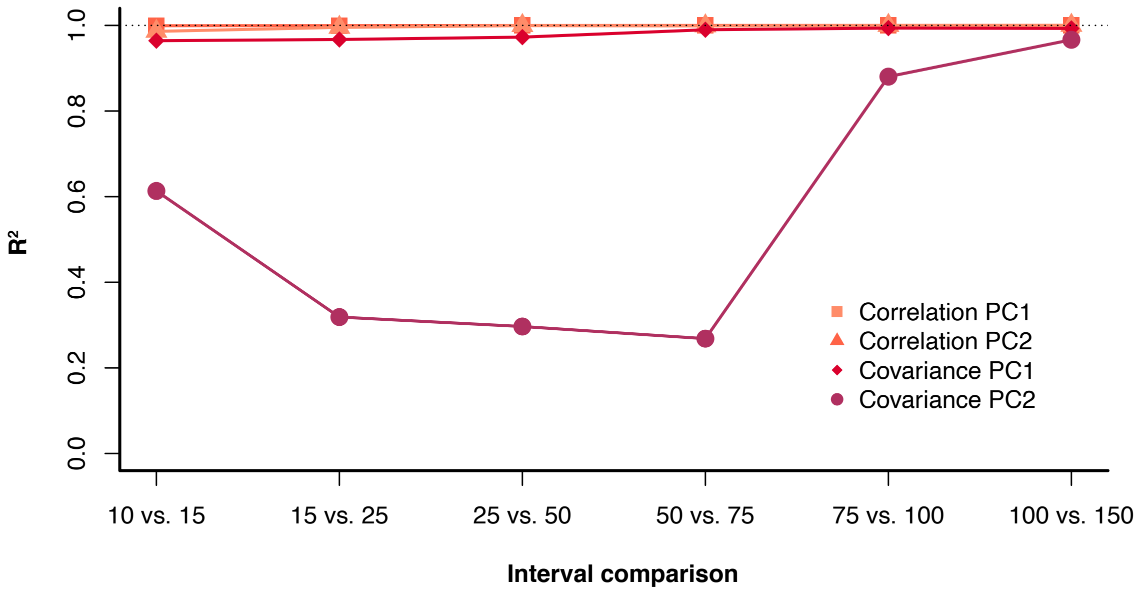

Fig. S3. Convergence of intervals. R^2 values of pairwise comparisons of PCAs using different number of intervals and either correlation or covariance matrices.

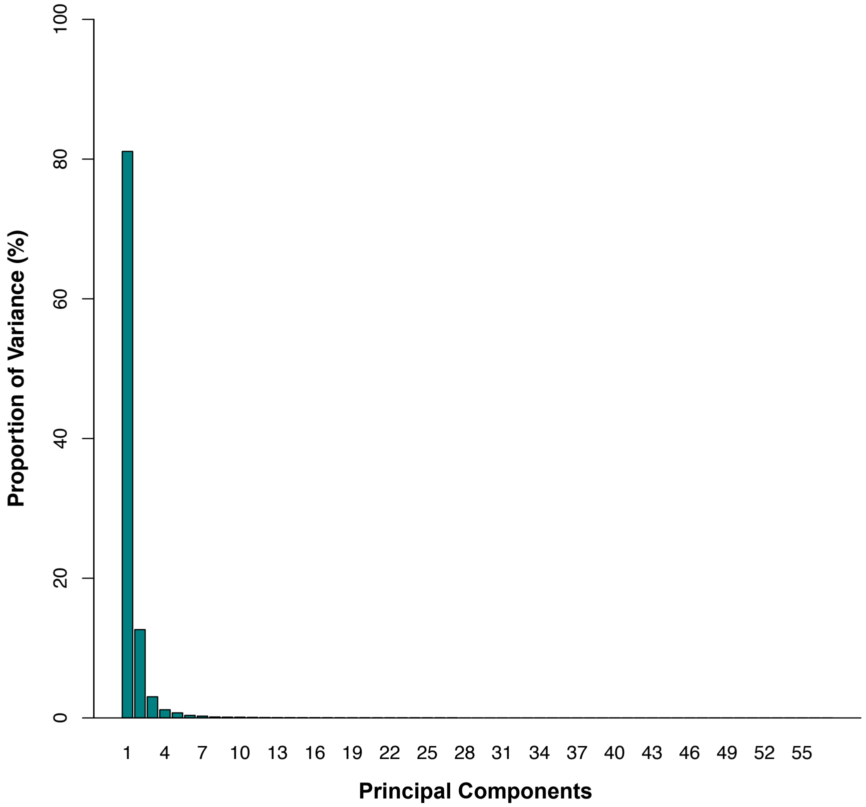

Fig. S4. Proportion of variance explained by each Principal Component resulting from the PCA on the stress intervals matrix.

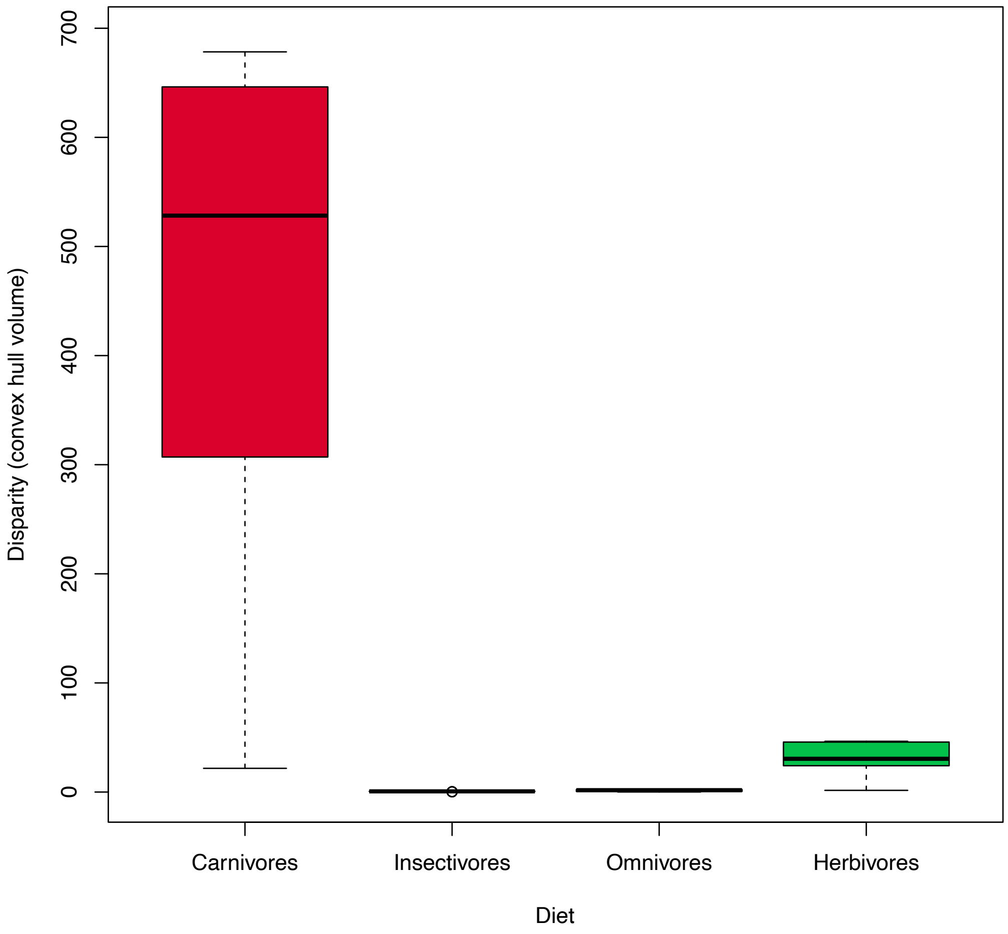

Fig. S5. Biomechanical disparity per dietary group. Disparity is measured as the convex hull volume of each group considering the first two PC axes of the biomechanical space. Disparity for durophages could not be calculated due to their small sample size.

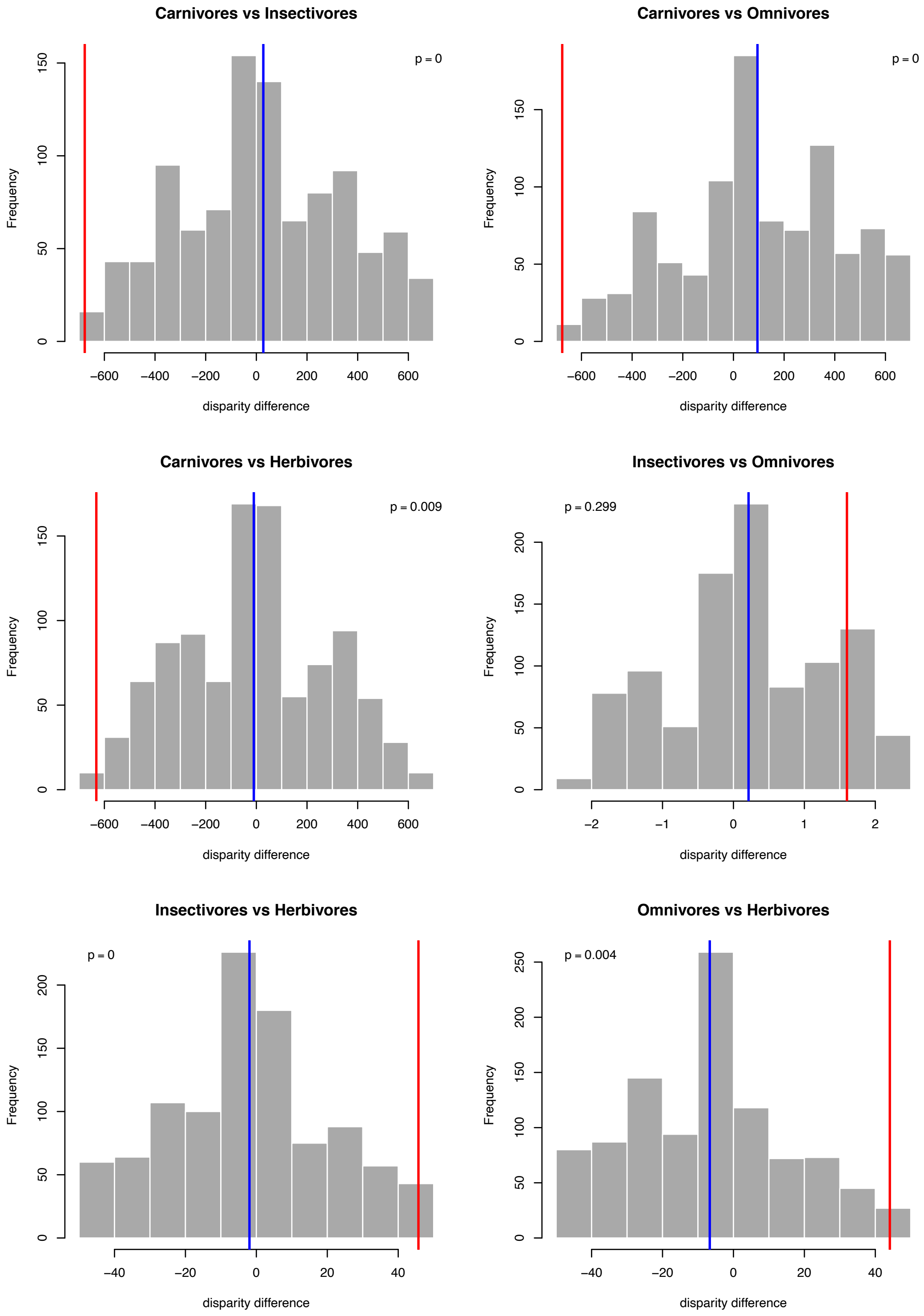

Fig. S6. Permutation test comparisons of biomechanical disparity between dietary groups. Lines show observed (red) and expected (blue) disparity differences and p-values (top of the histograms) indicate if differences are statistically significant when $p < 0.05$.

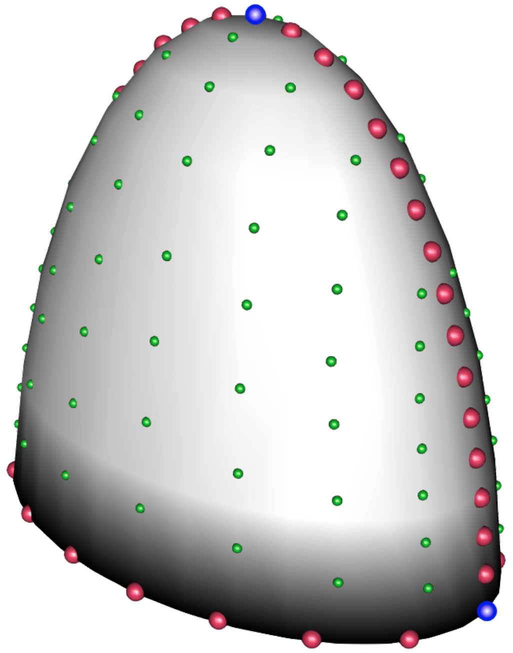

Fig. S7. Template used to transfer surface semi-landmarks to tooth models in mesiolabial view. Fixed landmarks are shown in blue, curve semi-landmarks in red and surface semi-landmarks in green.

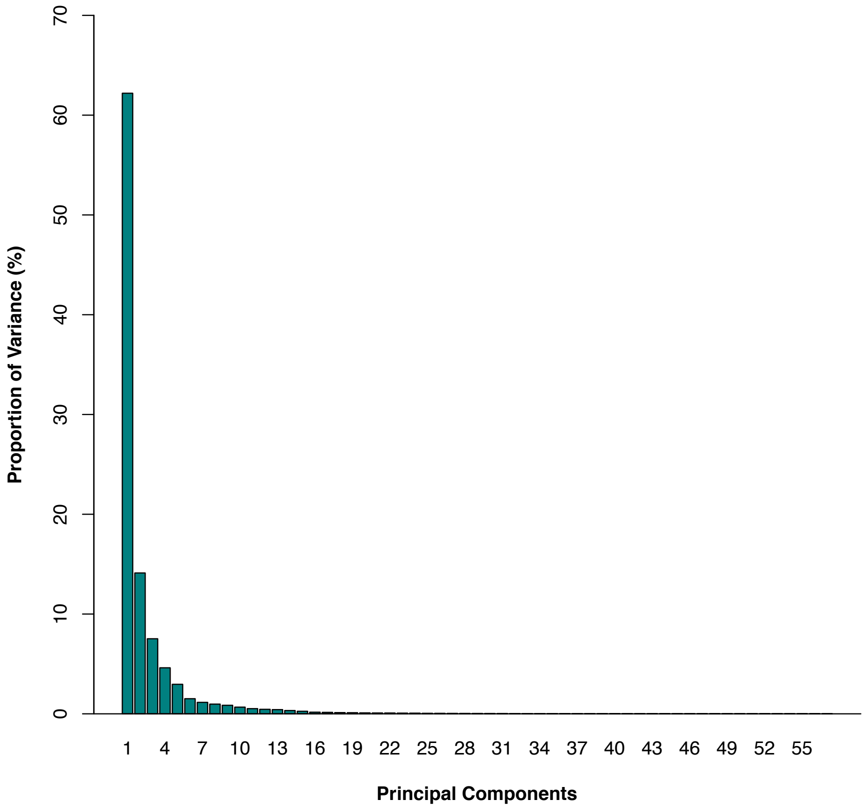

Fig. S8. Proportion of variance explained by each Principal Component resulting from the PCA on the Procrustes alignment.

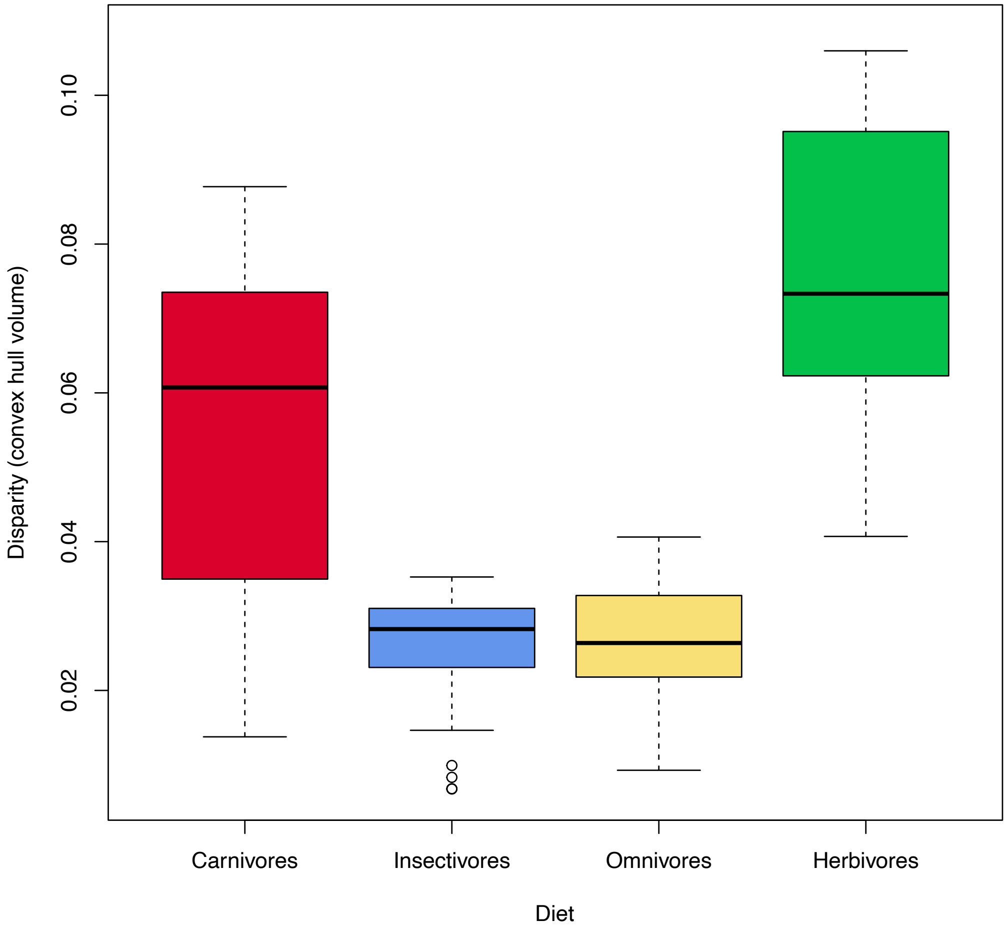

Fig. S9. Morphological disparity per dietary group. Disparity is measured as the convex hull volume of each group considering the first two PC axes of the morphological space. Disparity for durophages could not be calculated due to their small sample size.

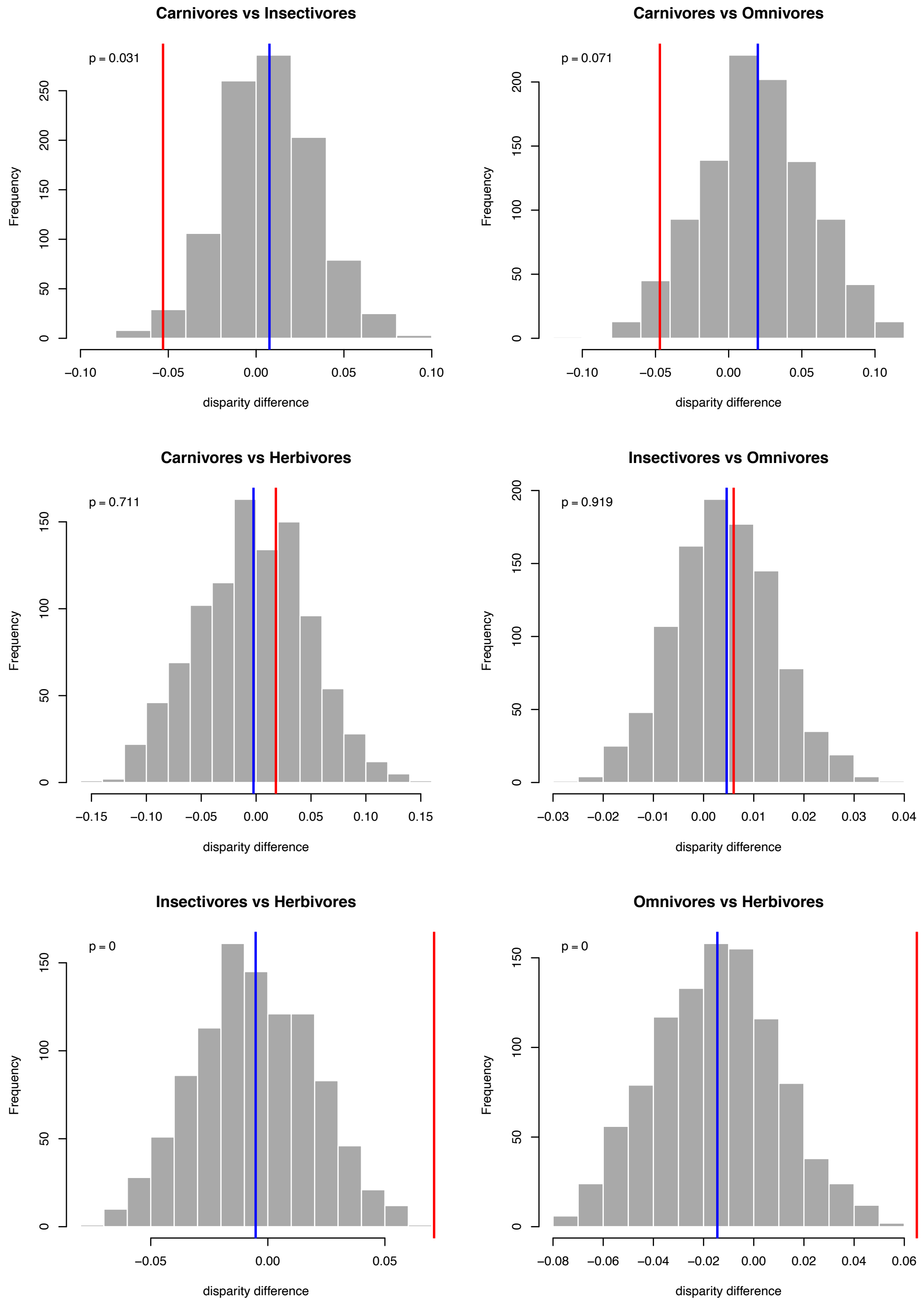

Fig. S10. Permutation test comparisons of morphological disparity between dietary groups. Lines show observed (red) and expected (blue) disparity differences and p-values (top left of the histograms) indicate if differences are statistically significant when $p < 0.05$.

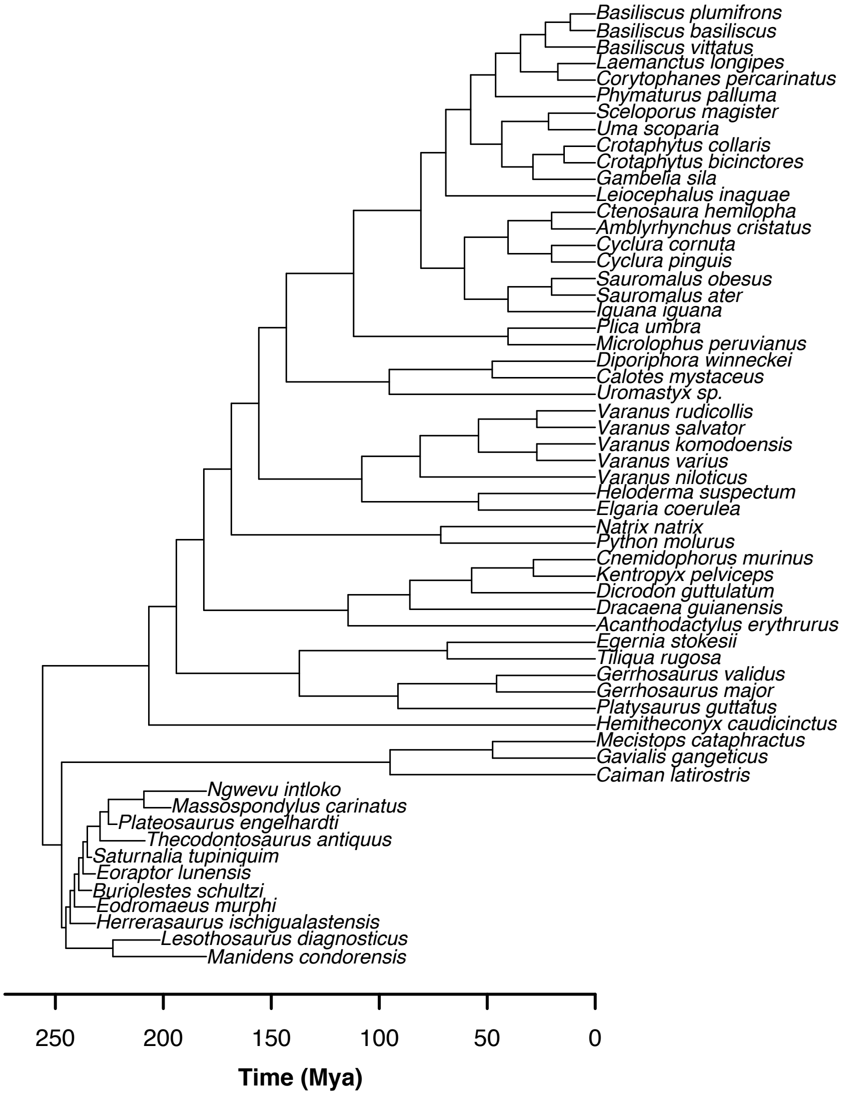

Fig. S11. Time-calibrated phylogeny of Sauropsida with the 58 taxa included in the study.

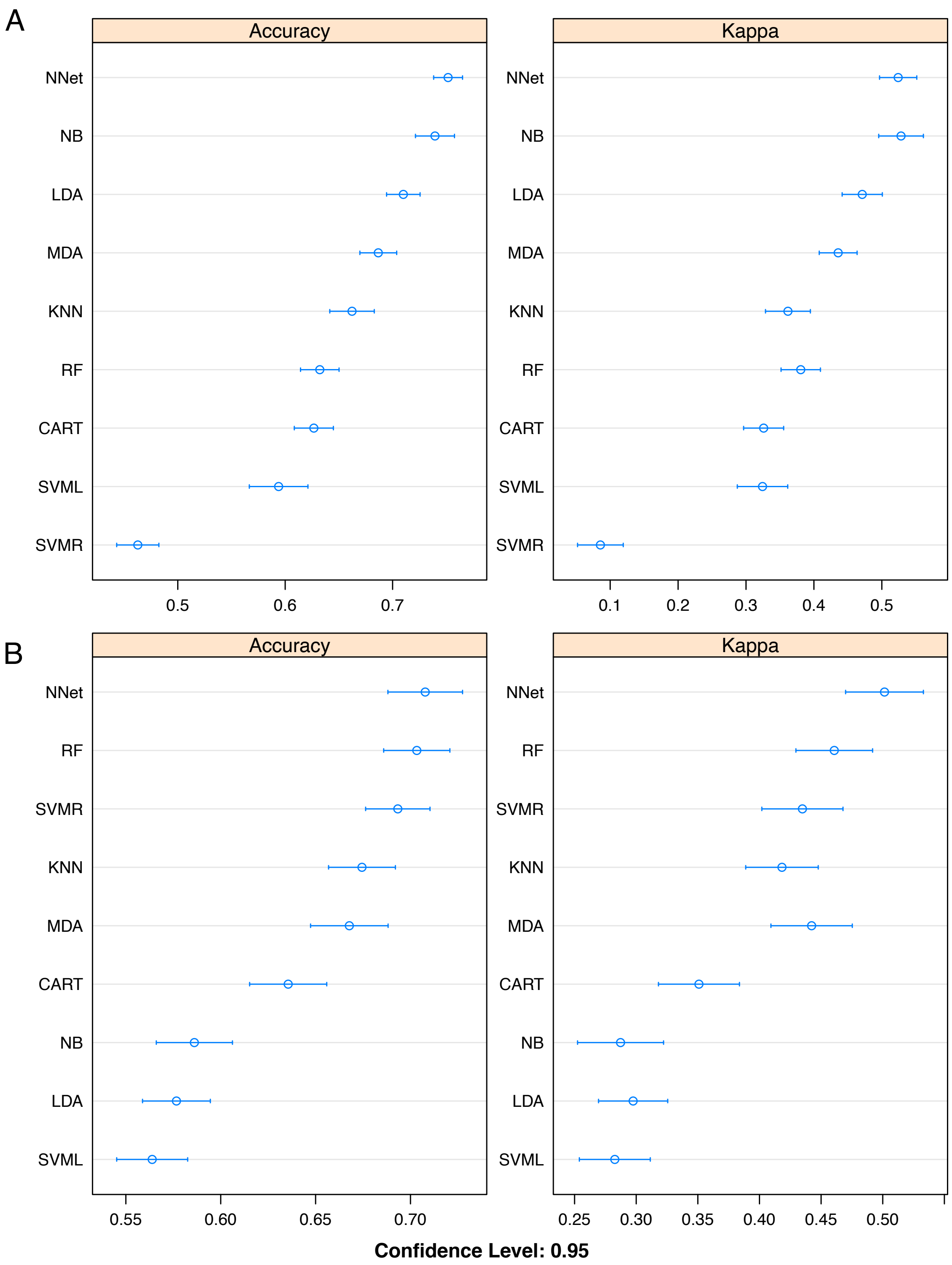

Fig. S12. Accuracy and Kohen's Kappa comparisons of nine machine learning algorithms tested on the (A) biomechanical and (B) morphological data.

Table S1. Specimen and data source information. Dietary information compiled from published quantitative datasets and ecological studies of lizards (113, 114), snakes (115, 116) and crocodylians (117–119). Predators were split into carnivores, insectivores and durophages depending on whether they preferentially prey on vertebrates, arthropods or hard-shelled animals, respectively (23).

Species	Specimen	Tooth position	Clade	Data type	Diet	Source	Author	Ref.
Crotaphytus bicinctores	CAS 200859	Right Dentary 19	Squamata	Surface model	Carnivore	MorphoSource	Keegan Melstrom	(23)
Gavialis gangeticus	UF H 118998	Left Dentary 10	Crocodylia	CT data	Carnivore	MorphoSource	David Blackburn	
Heloderma suspectum	CAS 159492	Right Dentary 4	Squamata	Surface model	Carnivore	MorphoSource	Keegan Melstrom	(23)
Mecistops cataphractus	TMM M-3529	Right Dentary 11	Crocodylia	CT data	Carnivore	MorphoSource	Jessica Maisano	
Natrix natrix	UMMZ H 65465	Right Dentary 9	Squamata	CT data	Carnivore	MorphoSource	Gregory Schneider	
Python molurus	UF H 190353	Right Dentary 14	Squamata	CT data	Carnivore	MorphoSource	David Blackburn	
Varanus komodoensis	TNHC 95803	Right Dentary 8	Squamata	CT data	Carnivore	MorphoSource	Jessica Maisano	
Varanus rudicollis	UCMP 137816	Right Dentary 10	Squamata	Surface model	Carnivore	MorphoSource	Keegan Melstrom	(23)
Varanus salvator	PIMUZ 7825	Right Dentary 3	Squamata	Surface model	Carnivore	MorphoSource	Eva Herbst	(120)
Varanus varius	MVZ 77092	Right Dentary 10	Squamata	Surface model	Carnivore	MorphoSource	Keegan Melstrom	(23)
Caiman latirostris	UMMZ H 155287	Left Dentary 18	Crocodylia	CT data	Durophage	MorphoSource	Gregory Schneider	
Dracaena guianensis	MVZ 79247	Right Dentary 9	Squamata	Surface model	Durophage	MorphoSource	Keegan Melstrom	(23)
Varanus niloticus	MVZ 68534	Right Dentary 9	Squamata	Surface model	Durophage	MorphoSource	Keegan Melstrom	(23)
Amblyrhynchus cristatus	MVZ 67721	Right Dentary 8	Squamata	Surface model	Herbivore	MorphoSource	Keegan Melstrom	(23)
Cyclura cornuta	UCMP 123055	Right Dentary 19	Squamata	Surface model	Herbivore	MorphoSource	Keegan Melstrom	(23)
Dicrodon guttulatum	MVZ 85400	Left Dentary 15	Squamata	Surface model	Herbivore	MorphoSource	Keegan Melstrom	(23)
Egernia stokesii	MCZ R-33105	Right Dentary 14	Squamata	Surface model	Herbivore	MorphoSource	Keegan Melstrom	(23)
Iguana iguana	UMNH 8084	Right Dentary 13	Squamata	Surface model	Herbivore	MorphoSource	Keegan Melstrom	(23)
Phymaturus palluma	MVZ 137647	Right Dentary 13	Squamata	Surface model	Herbivore	MorphoSource	Keegan Melstrom	(23)
Sauromalus ater	MVZ 100404	Right Dentary 13	Squamata	Surface model	Herbivore	MorphoSource	Keegan Melstrom	(23)
Sauromalus obesus	UCMP 137811	Right Dentary 17	Squamata	Surface model	Herbivore	MorphoSource	Keegan Melstrom	(23)
Tiliqua rugosa	MCZ R-24456	Right Dentary 10	Squamata	Surface model	Herbivore	MorphoSource	Keegan Melstrom	(23)
Uromastyx sp.	UCMP 118912	Right Dentary 10	Squamata	Surface model	Herbivore	MorphoSource	Keegan Melstrom	(23)
Basiliscus vittatus	UCMP 137748	Right Dentary 18	Squamata	Surface model	Insectivore	MorphoSource	Keegan Melstrom	(23)
Calotes mystaceus	CAS 243761	Right Dentary 14	Squamata	Surface model	Insectivore	MorphoSource	Keegan Melstrom	(23)
Corytophanes percarinatus	UCMP 123057	Right Dentary 19	Squamata	Surface model	Insectivore	MorphoSource	Keegan Melstrom	(23)
Crotaphytus collaris	UCMP 141134	Right Dentary 15	Squamata	Surface model	Insectivore	MorphoSource	Keegan Melstrom	(23)
Elgaria coerulea	CAS 216644	Right Dentary 19	Squamata	Surface model	Insectivore	MorphoSource	Keegan Melstrom	(23)
Gambelia sila	CAS 141318	Right Dentary 17	Squamata	Surface model	Insectivore	MorphoSource	Keegan Melstrom	(23)

Hemitheconyx caudicinctus	CAS uncatalogued	Right Dentary 23	Squamata	Surface model	Insectivore	MorphoSource	Keegan Melstrom	(23)
Kentropyx pelviceps	MVZ 174866	Right Dentary 18	Squamata	Surface model	Insectivore	MorphoSource	Keegan Melstrom	(23)
Laemanctus longipes	UCMP 129880	Right Dentary 18	Squamata	Surface model	Insectivore	MorphoSource	Keegan Melstrom	(23)
Plica umbra	CAS 93242	Right Dentary 18	Squamata	Surface model	Insectivore	MorphoSource	Keegan Melstrom	(23)
Sceloporus magister	CAS 200862	Right Dentary 20	Squamata	Surface model	Insectivore	MorphoSource	Keegan Melstrom	(23)
Acanthodactylus erythrurus	SDNHM 65156	Right Dentary 18	Squamata	Surface model	Omnivore	MorphoSource	Keegan Melstrom	(23)
Basiliscus basiliscus	MVZ 79579	Right Dentary 14	Squamata	Surface model	Omnivore	MorphoSource	Keegan Melstrom	(23)
Basiliscus plumifrons	MCZ R-19487	Right Dentary 17	Squamata	Surface model	Omnivore	MorphoSource	Keegan Melstrom	(23)
Cnemidophorus murinus	OU 39632	Right Dentary 10	Squamata	Surface model	Omnivore	MorphoSource	Keegan Melstrom	(23)
Ctenosaura hemilopha	CAS 46399	Right Dentary 13	Squamata	Surface model	Omnivore	MorphoSource	Keegan Melstrom	(23)
Cyclura pinguis	KU 272292	Left Dentary 22	Squamata	Surface model	Omnivore	MorphoSource	Keegan Melstrom	(23)
Diporiphora winneckei	MCZ R-35222	Right Dentary 9	Squamata	Surface model	Omnivore	MorphoSource	Keegan Melstrom	(23)
Gerrhosaurus major	UCMP 137878	Right Dentary 16	Squamata	Surface model	Omnivore	MorphoSource	Keegan Melstrom	(23)
Gerrhosaurus validus	MCZ R-50973	Right Dentary 21	Squamata	Surface model	Omnivore	MorphoSource	Keegan Melstrom	(23)
Leiocephalus inaguae	MCZ R-154263	Right Dentary 20	Squamata	Surface model	Omnivore	MorphoSource	Keegan Melstrom	(23)
Microlophus peruvianus	UCMP 141136	Right Dentary 17	Squamata	Surface model	Omnivore	MorphoSource	Keegan Melstrom	(23)
Platysaurus guttatus	MCZ R-44415	Right Dentary 16	Squamata	Surface model	Omnivore	MorphoSource	Keegan Melstrom	(23)
Uma scoparia	CAS 42076	Right Dentary 24	Squamata	Surface model	Omnivore	MorphoSource	Keegan Melstrom	(23)
Manidens condorensis	MPEF-PV 3211	Right Dentary 9	Dinosauria	CT data	Unknown	From author	Marcos Becerra	(49)
Lesothosaurus diagnosticus	BP/1/7853	Left Dentary 14	Dinosauria	CT data	Unknown	From author	Lara Sciscio	(61)
Eodromaeus murphi	PVSJ 560	Right Dentary ?	Dinosauria	CT data	Unknown	From author	Paul Barrett, David Button, Ricardo Martínez, Laura Porro	
Herrerasaurus ischigualastensis	PVSJ 407	Right Dentary 11	Dinosauria	CT data	Unknown	From author	Paul Barrett, David Button, Ricardo Martínez, Laura Porro	
Buriolestes schultzi	CAPPA/UFSM 0035	Right Maxillary 8	Dinosauria	CT data	Unknown	From author	Rodrigo Müller	(56)
Eoraptor lunensis	PVSJ 512	Left Maxillary 6	Dinosauria	CT data	Unknown	Digimorph	Jessica Maisano	
Saturnalia tupiniquim	MCP-3845-PV	Right Dentary 9?	Dinosauria	CT data	Unknown	From author	Mario Bronzati	(20)
Thecodontosaurus antiquus	BRSUG 28221	Left Maxillary ?	Dinosauria	CT data	Unknown	From author	Antonio Ballell	(40)
Plateosaurus engelhardti	MB.R.1937	Left Dentary 14	Dinosauria	CT data	Unknown	From author	Lawrence Witmer	(47)
Massospondylus carinatus	BP/1/5241	Left Maxillary 8	Dinosauria	CT data	Unknown	From author	Kimberley Chapelle	(96)
Ngwevu intloko	BP/1/4779	Right Maxillary 11	Dinosauria	CT data	Unknown	From author	Kimberley Chapelle	(39)

Table S2. Details of 2D, 3D meshes and original surface area and volume of tooth models.

Model	2D mesh	3D mesh		Tooth surface area (mm^2)	Tooth volume (mm^3)
	No. triangles	No. elements	No. nodes		
Acanthodactylus erythrurus	121610	1443842	212665	8.205E-05	5.373E-08
Amblyrhynchus cristatus	125012	1273969	183360	3.656E-04	3.914E-07
Basiliscus basiliscus	124112	1369388	199576	4.260E-04	5.851E-07
Basiliscus plumifrons	122612	1347262	196160	4.735E-04	7.052E-07
Basiliscus vittatus	119980	1292079	187555	1.815E-04	1.614E-07
Buriolestes schultzi	122458	1334852	194036	3.883E-04	5.038E-07
Caiman latirostris	122950	1367401	199419	8.243E-03	5.577E-05
Calotes mystaceus	125158	1444632	274444	1.373E-04	9.390E-07
Cnemidophorus murinus	120956	1484242	219617	9.063E-05	6.382E-08
Corytophanes percarinatus	120138	1350974	197456	3.779E-04	4.727E-07
Crotaphytus bicinctores	122808	1492185	220402	2.029E-04	1.995E-07
Crotaphytus collaris	122264	1451999	213902	3.864E-04	5.283E-07
Ctenosaura hemilopha	122524	1342753	195501	7.170E-05	4.026E-08
Cyclura cornuta	122938	1345600	195879	4.255E-04	5.398E-07
Cyclura pinguis	121548	1306602	189644	2.554E-04	2.698E-07
Dicrodon guttulatum	121800	1461539	215654	1.620E-04	1.449E-07
Diporiphora winneckei	121556	1438749	211761	1.490E-04	1.197E-07
Dracaena guianensis	117266	1481481	220155	2.034E-02	2.023E-04
Egernia stokesii	121760	1420876	208723	5.371E-05	2.986E-08
Elgaria coerulea	123124	1401739	205190	2.038E-04	1.944E-07
Eodromaeus murphi	118936	1233313	177841	1.449E-03	3.090E-06
Eoraptor lunensis	120432	1284714	186275	3.712E-03	1.360E-05
Gambelia sila	120162	1391578	204212	3.219E-04	3.915E-07
Gavialis gangeticus	123510	1255332	180524	2.197E-02	1.784E-04
Gerrhosaurus major	117818	1376988	202353	6.130E-04	1.170E-06
Gerrhosaurus validus	121086	1418604	208485	9.711E-05	7.072E-08
Heloderma suspectum	122088	1217125	174527	2.273E-03	5.403E-06
Hemitheconyx caudicinctus	121620	1417703	208288	4.086E-05	1.978E-08
Herrerasaurus ischigualastensis	119614	1259931	182248	1.116E-02	6.681E-05
Iguana iguana	122124	1300704	188477	5.405E-04	7.551E-07
Kentropyx pelviceps	119674	1340582	195802	5.030E-04	7.645E-07
Laemanctus longipes	118226	1317744	192290	3.342E-04	4.393E-07
Leiocephalus inaguae	121994	1429824	210213	6.427E-04	1.225E-06
Lesothosaurus diagnosticus	125788	1363519	198106	2.150E-03	6.799E-06
Manidens condorensis	120478	1200434	171777	3.123E-03	9.301E-06
Massospondylus carinatus	118766	1224601	176500	9.619E-03	5.640E-05
Mecistops cataphractus	118966	1282708	186283	1.945E-02	1.852E-04
Microlophus peruvianus	121658	1398018	204870	5.723E-05	2.951E-08

Natrix natrix	124280	1320729	190969	1.248E-04	7.861E-08
Ngwevu intloko	119302	1230512	177435	6.945E-03	3.465E-05
Phymaturus palluma	123582	1347838	196029	7.486E-05	4.280E-08
Plateosaurus engelhardti	121198	1266918	183008	2.011E-02	1.748E-04
Platysaurus guttatus	122162	1423457	209163	6.437E-05	3.841E-08
Plica umbra	120774	1399857	205418	1.216E-04	9.358E-08
Python molurus	124642	1303669	188257	1.890E-03	4.606E-06
Saturnalia tupiniquim	121544	1305620	189435	1.725E-03	4.765E-06
Sauromalus ater	123370	1356743	197672	2.237E-04	2.145E-07
Sauromalus obesus	124300	1410188	206282	3.109E-04	3.545E-07
Sceloporus magister	121002	1406836	206546	1.584E-04	1.425E-07
Thecodontosaurus antiquus	124202	1287487	185759	1.855E-03	4.682E-06
Tiliqua rugosa	122774	1530451	226861	2.144E-03	6.739E-06
Uma scoparia	120844	1442058	212523	6.069E-05	3.276E-08
Uromastyx sp	122304	1508648	223325	1.815E-04	1.548E-07
Varanus komodoensis	120936	1236894	178019	1.523E-02	1.046E-04
Varanus niloticus	121766	1413187	207495	7.054E-03	4.964E-05
Varanus rudicollis	121920	1273016	183866	2.640E-03	7.985E-06
Varanus salvator	121042	1285660	186189	5.816E-03	2.426E-05
Varanus varius	121722	1299564	188373	4.537E-03	1.812E-05

Table S3. Constraint test. Von Mises stress mesh-weighted arithmetic mean (MPa) with varying number of constrained nodes around the base of the tooth FE model of *Iguana iguana*, position of the reference point (base or root) and error between observations.

Nodes	Base constraint MWAM	Root constraint MWAM	Error %
20	3206235.68	3206235.68	0.797
40	3180873.22	3180873.22	0.462
60	3166249.23	3166249.23	0.230
80	3158976.04	3158976.04	0.496
All (378)	3143387.61	3143387.61	NA

Table S4. Convergence test. Von Mises stress mesh-weighted arithmetic mean (MPa) with varying number of tetrahedral elements and corresponding mean element size in mm, with error between observations.

Element size	No. elements	MWAM	Error %
0.05	9914170	3219143.48	0.437
0.075	6656992	3233270.41	1.206
0.1	3296634	3194732.59	1.842
0.125	1983843	3136949.88	0.957
0.15	1300704	3107215.41	2.975
0.175	910179	3202503.45	2.697
0.2	663545	3118399.04	NA

Table S5. Minimum node ages of eight sauropsid clades used to constrain the time calibration of the phylogeny.

Clade	Taxon 1	Taxon 2	Minimum age (Mya)	Ref.
Sauropsida	*Gavialis gangeticus*	*Basiliscus basiliscus*	255.9	(101)
Archosauria	*Gavialis gangeticus*	*Massospondylus carinatus*	247.1	(101)
Crocodylia	*Gavialis gangeticus*	*Caiman latirostris*	95	(98)
Squamata	*Hemitheconyx caudicinctus*	*Basiliscus basiliscus*	206.7	(101)
Scincoidea	*Egernia stokesii*	*Gerrhosaurus major*	137.02	(102)
Anguimorpha	*Heloderma suspectum*	*Varanus varius*	108.09	(102)
Iguania	*Uromastyx sp.*	*Basiliscus basiliscus*	143.05	(102)
Euiguana	*Iguana iguana*	*Basiliscus basiliscus*	80.71	(102)

Table S6. Phylogenetic multivariate regression (PGLS) of Von Mises stress MWAM on tooth size (log tooth Centroid Size).

	df	SS.obs	MS	F	P.val	Rsq
**log.Csize**	1	112859.9	112859.86	2.112862	0.3216783	0.0363579
**Residual**	56	2991275.9	53415.64			

Table S7. Phylogenetic multivariate regression (PGLS) of Von Mises stress MWAM on tooth size (log tooth surface area).

	df	SS.obs	MS	F	P.val	Rsq
**log.sa**	1	63387.38	63387.38	1.167375	0.4735265	0.0204203
**Residual**	56	3040748.35	54299.08			

Table S8. Phylogenetic multivariate regression (PGLS) of Von Mises stress MWAM on tooth size (log tooth volume).

	df	SS.obs	MS	F	P.val	Rsq
**log.vol**	1	37463.22	37463.22	0.6841096	0.5734266	0.01206881
**Residual**	56	3066672.51	54762.01			

Table S9. Phylogenetic multivariate regression (PGLS) of Von Mises stress intervals on tooth size (log tooth Centroid Size).

	df	SS.obs	MS	F	P.val	Rsq
**log.Csize**	1	7.235924	7.235924	1.408326	0.5444555	0.02453174
**Residual**	56	287.725839	5.137961			

Table S10. Phylogenetic multivariate regression (PGLS) of Von Mises stress intervals on tooth size (log tooth surface area).

	df	SS.obs	MS	F	P.val	Rsq
**log.sa**	1	4.866353	4.866353	0.9394005	0.7222777	0.01649825
**Residual**	56	290.09541	5.180275			

Table S11. Phylogenetic multivariate regression (PGLS) of Von Mises stress intervals on tooth size (log tooth volume).

	df	SS.obs	MS	F	P.val	Rsq
**log.vol**	1	4.012408	4.012408	0.7722816	0.7542458	0.01360315
**Residual**	56	290.949356	5.195524			

Table S12. Phylogenetic multivariate regression (PGLS) of Procrustes coordinates on tooth size (log tooth Centroid Size).

	Df	SS	MS	Rsq	F	Z	Pr(>F)
**log.Csize**	1	0.002234	0.00223406	0.03928	2.2896	1.7286	0.044*
**Residuals**	56	0.054641	0.00097573	0.96072			
**Total**	57	0.056875					

Table S13. Phylogenetic multivariate regression (PGLS) of Procrustes coordinates on tooth size (log tooth surface area).

	Df	SS	MS	Rsq	F	Z	Pr(>F)
**log.sa**	1	0.001997	0.00199688	0.03511	2.0377	1.5029	0.066
**Residuals**	56	0.054878	0.00097997	0.96489			
**Total**	57	0.056875					

Table S14. Phylogenetic multivariate regression (PGLS) of Procrustes coordinates on tooth size (log tooth volume).

	Df	SS	MS	Rsq	F	Z	Pr(>F)
**log.vol**	1	0.00186	0.00185955	0.0327	1.8928	1.4	0.084
**Residuals**	56	0.055015	0.00098242	0.9673			
**Total**	57	0.056875					

Table S15. Procrustes ANOVA comparing tooth Procrustes coordinates across dietary categories.

	Df	SS	MS	Rsq	F	Z	Pr(<F)
**diets**	4	1.0842	0.27105	0.35224	5.7097	3.6162	0.001**
**Residuals**	42	1.9938	0.047472	0.64776			
**Total**	46	3.078					

Table S16. Phylogenetic Procrustes ANOVA comparing tooth Procrustes coordinates across dietary categories.

	Df	SS	MS	Rsq	F	Z	Pr(<F)
**diets**	4	0.005516	0.0013789	0.16908	2.1367	2.0854	0.016*
**Residuals**	42	0.027105	0.00064535	0.83092			
**Total**	46	0.03262					

Table S17. Pairwise PERMANOVA of biomechanical and morphological PC coordinates that explain 90% of the variance, dividing the extant sample in five dietary categories. Values indicate Bonferroni corrected p-values. Statistically significant values are marked in bold.

**Biomechanical (2 PCs)**					
	Omnivore	Herbivore	Insectivore	Durophage	Carnivore
Omnivore		0.366	1	1	**0.002**
Herbivore	0.366		0.309	1	**0.029**
Insectivore	1	0.309		0.412	**0.001**
Durophage	1	1	0.412		0.586
Carnivore	**0.002**	**0.029**	**0.001**	0.586	
**Morphological (5 PCs)**					
	Omnivore	Herbivore	Insectivore	Durophage	Carnivore
Omnivore		1	1	1	**0.001**
Herbivore	1		1	1	**0.001**
Insectivore	1	1		1	**0.001**
Durophage	1	1	1		0.076
Carnivore	**0.001**	**0.001**	**0.001**	0.076	

Table S18. Pairwise PERMANOVA of biomechanical and morphological PC coordinates that explain 90% of the variance, dividing the extant sample in three dietary categories. Values indicate Bonferroni corrected p-values. Statistically significant values are marked in bold.

**Biomechanical (2 PCs)**			
	Omnivore	Herbivore	Carnivore
Omnivore		**0.0135**	**0.0003**
Herbivore	**0.0135**		**0.0117**
Carnivore	**0.0003**	**0.0117**	
**Morphological (5 PCs)**			
	Omnivore	Herbivore	Carnivore
Omnivore		1	**0.0003**
Herbivore	1		**0.0015**
Carnivore	**0.0003**	**0.0015**	

Table S19. Accuracy and Kohen's Kappa comparisons of nine machine learning algorithms tested on the biomechanical data.

Accuracy						
	**Minimum**	**1st Quartile**	**Median**	**Mean**	**3rd Quartile**	**Maximum**
**CART**	0.111	0.556	0.667	0.627	0.667	0.889
**LDA**	0.444	0.667	0.667	0.710	0.778	1.000
**MDA**	0.222	0.667	0.667	0.687	0.778	1.000
**SVML**	0.111	0.444	0.667	0.594	0.778	0.889
**SVMR**	0.111	0.333	0.444	0.463	0.556	0.778
**KNN**	0.000	0.667	0.667	0.662	0.778	1.000
**NB**	0.333	0.667	0.778	**0.739**	0.778	1.000
**RF**	0.333	0.556	0.667	0.632	0.667	0.889
**NNet**	0.444	0.667	0.778	**0.752**	0.778	1.000
**Kappa**						
	**Minimum**	**1st Quartile**	**Median**	**Mean**	**3rd Quartile**	**Maximum**
**CART**	-0.400	0.200	0.357	0.326	0.438	0.813
**LDA**	-0.154	0.357	0.438	0.471	0.600	1.000
**MDA**	-0.200	0.333	0.400	0.436	0.600	1.000
**SVML**	-0.500	0.143	0.308	0.324	0.571	0.813
**SVMR**	-0.500	-0.070	0.118	0.086	0.250	0.625
**KNN**	-0.500	0.308	0.308	0.362	0.571	1.000
**NB**	-0.286	0.357	0.571	**0.528**	0.647	1.000
**RF**	-0.125	0.250	0.400	0.381	0.507	0.824
**NNet**	-0.071	0.357	0.571	**0.524**	0.600	1.000

Table S20. Accuracy and Kohen's Kappa comparisons of nine machine learning algorithms tested on the morphological data.

Accuracy						
	**Min.**	**1st Q**	**Median**	**Mean**	**3rd Q**	**Max.**
**CART**	0.222	0.556	0.667	0.636	0.778	0.889
**LDA**	0.111	0.444	0.556	0.577	0.667	0.889
**MDA**	0.333	0.556	0.667	0.668	0.778	1.000
**SVML**	0.222	0.444	0.556	0.564	0.667	0.889
**SVMR**	0.222	0.667	0.667	0.693	0.778	1.000
**KNN**	0.222	0.556	0.667	0.674	0.778	0.889
**NB**	0.222	0.444	0.556	0.586	0.667	0.889
**RF**	0.333	0.667	0.667	0.703	0.778	1.000
**NNet**	0.333	0.667	0.667	**0.708**	0.778	1.000
**Kappa**						
	**Min.**	**1st Q**	**Median**	**Mean**	**3rd Q**	**Max.**
**CART**	-0.286	0.200	0.345	0.351	0.571	0.800
**LDA**	-0.333	0.143	0.333	0.298	0.471	0.824
**MDA**	-0.125	0.283	0.471	0.442	0.625	1.000
**SVML**	-0.400	0.118	0.250	0.283	0.400	0.824
**SVMR**	-0.313	0.308	0.419	0.435	0.600	1.000
**KNN**	-0.125	0.283	0.400	0.418	0.571	0.824
**NB**	-0.400	0.118	0.294	0.287	0.446	0.824
**RF**	-0.125	0.308	0.400	0.461	0.600	1.000
**NNet**	0.000	0.383	0.498	**0.501**	0.625	1.000

Table S21. Diet prediction and dietary class probability of early dinosaurs. The classifications are based on the final machine learning model for the biomechanical and morphological data.

Species	Prediction	Carnivore probability	Herbivore probability	Omnivore probability
**Biomechanical classification**				
Manidens condorensis	Carnivore	0.953	0.046	0.001
Lesothosaurus diagnosticus	Omnivore	0.001	0.093	0.906
Herrerasaurus ischigualastensis	Carnivore	0.861	0.138	0.001
Eodromaeus murphi	Carnivore	0.997	0.002	0.001
Buriolestes schultzi	Carnivore	0.574	0.425	0.001
Eoraptor lunensis	Carnivore	0.959	0.040	0.001
Saturnalia tupiniquim	Omnivore	0.006	0.350	0.644
Thecodontosaurus antiquus	Herbivore	0.218	0.782	0.000
Plateosaurus engelhardti	Herbivore	0.010	0.545	0.445
Massospondylus carinatus	Herbivore	0.022	0.945	0.034
Ngwevu intloko	Herbivore	0.024	0.972	0.004
**Morphological classification**				
Manidens condorensis	Herbivore	0.004	0.996	0.000
Lesothosaurus diagnosticus	Herbivore	0.016	0.919	0.065
Herrerasaurus ischigualastensis	Carnivore	0.954	0.040	0.006
Eodromaeus murphi	Carnivore	0.958	0.018	0.024
Buriolestes schultzi	Carnivore	0.971	0.001	0.028
Eoraptor lunensis	Carnivore	0.586	0.412	0.002
Saturnalia tupiniquim	Carnivore	0.855	0.123	0.022
Thecodontosaurus antiquus	Herbivore	0.126	0.871	0.003
Plateosaurus engelhardti	Herbivore	0.013	0.987	0.000
Massospondylus carinatus	Herbivore	0.036	0.963	0.001
Ngwevu intloko	Herbivore	0.020	0.980	0.000

REFERENCES AND NOTES

1. P. M. Barrett, E. J. Rayfield, Ecological and evolutionary implications of dinosaur feeding behaviour. *Trends Eco. Evo.* **21**, 217–224 (2006).

2. P. M. Barrett, R. J. Butler, S. J. Nesbitt, The roles of herbivory and omnivory in early dinosaur evolution. *Earth Environ. Sci. Trans. R. Soc. Edinb.* **101**, 383–396 (2011).

3. L. E. Zanno, P. J. Makovicky, Herbivorous ecomorphology and specialization patterns in theropod dinosaur evolution. *Proc. Natl. Acad. Sci. U.S.A.* **108**, 232–237 (2011).

4. P. M. Barrett, Paleobiology of herbivorous dinosaurs. *Annu. Rev. Earth Planet. Sci.* **42**, 207–230 (2014).

5. J. A. MacLaren, P. S. L. Anderson, P. M. Barrett, E. J. Rayfield, Herbivorous dinosaur jaw disparity and its relationship to extrinsic evolutionary drivers. *Paleobiology* **43**, 15–33 (2017).

6. D. J. Button, L. E. Zanno, Repeated evolution of divergent modes of herbivory in non-avian dinosaurs. *Curr. Biol.* **30**, 158–168.e4 (2019).

7. M. G. Baron, D. B. Norman, P. M. Barrett, A new hypothesis of dinosaur relationships and early dinosaur evolution. *Nature* **543**, 501–506 (2017).

8. M. C. Langer, M. D. Ezcurra, O. W. M. Rauhut, M. J. Benton, F. Knoll, B. W. McPhee, F. E. Novas, D. Pol, S. L. Brusatte, Untangling the dinosaur family tree. *Nature* **551**, E1–E3 (2017).

9. R. T. Müller, M. S. Garcia, A paraphyletic 'Silesauridae' as an alternative hypothesis for the initial radiation of ornithischian dinosaurs. *Biol. Lett.* **16**, 20200417 (2020).

10. P. M. Barrett, "Prosauropods and iguanas: Speculation on the diets of extinct reptiles" in *The Evolution of Herbivory in Terrestrial Vertebrates: Perspectives From The Fossil Record*, H.-D. Sues, Ed. (Cambridge Univ. Press, 2000), pp. 42–78.

11. S. F. Cabreira, A. W. A. Kellner, S. Dias-da-Silva, L. Roberto da Silva, M. Bronzati, J. C. de Almeida Marsola, R. T. Müller, J. . S. Bittencourt, B. J.'A. Batista, T. Raugust, R. Carrilho, A.

Brodt, M. C. Langer, A unique Late Triassic dinosauromorph assemblage reveals dinosaur ancestral anatomy and diet. *Curr. Biol.* **26**, 3090–3095 (2016).

12. P. C. Sereno, F. E. Novas, The skull and neck of the basal theropod *Herrerasaurus ischigualastensis*. *J. Vertebr. Paleontol.* **13**, 451–476 (1994).

13. R. N. Martinez, P. C. Sereno, O. A. Alcober, C. E. Colombi, P. R. Renne, I. P. Montañez, B. S. Currie, A basal dinosaur from the dawn of the dinosaur era in Southwestern Pangaea. *Science* **331**, 206–210 (2011).

14. P. M. Galton, Diet of prosauropod dinosaurs from the Late Triassic and Early Jurassic. *Lethaia* **18**, 105–123 (1985).

15. P. C. Sereno, *Lesothosaurus*, "fabrosaurids," and the early evolution of Ornithischia. *J. Vertebr. Paleontol.* **11**, 168–197 (1991).

16. P. C. Sereno, The origin and evolution of dinosaurs. *Annu. Rev. Earth Planet. Sci.* **25**, 435–489 (1997).

17. R. N. Martínez, O. A. Alcober, A basal sauropodomorph (Dinosauria: Saurischia) from the Ischigualasto formation (Triassic, Carnian) and the early evolution of Sauropodomorpha. *PLOS ONE* **4**, e4397 (2009).

18. R. J. Butler, The anatomy of the basal ornithischian dinosaur *Eocursor parvus* from the lower Elliot Formation (Late Triassic) of South Africa. *Zool. J. Linn. Soc.* **160**, 648–684 (2010).

19. P. C. Sereno, R. N. Martínez, O. A. Alcober, Osteology of *Eoraptor lunensis* (Dinosauria, Sauropodomorpha). *J. Vertebr. Paleontol.* **32**, 83–179 (2012).

20. M. Bronzati, R. T. Müller, M. C. Langer, Skull remains of the dinosaur *Saturnalia tupiniquim* (Late Triassic, Brazil): With comments on the early evolution of sauropodomorph feeding behaviour. *PLOS ONE* **14**, e0221387 (2019).

21. M. Qvarnström, J. V. Wernström, R. Piechowski, M. Tałanda, P. E. Ahlberg, G. Niedźwiedzki, Beetle-bearing coprolites possibly reveal the diet of a Late Triassic dinosauriform. *R. Soc. Open Sci.* **6**, 181042 (2019).

22. P. W. Lucas, *Dental Functional Morphology: How Teeth Work* (Cambridge Univ. Press, 2010).

23. K. M. Melstrom, The relationship between diet and tooth complexity in living dentigerous saurians. *J. Morphol.* **278**, 500–522 (2017).

24. F. Lafuma, I. J. Corfe, J. Clavel, N. Di-Poi, Multiple evolutionary origins and losses of tooth complexity in squamates. *Nat. Commun.* **12**, 6001 (2021).

25. A. R. Evans, G. D. Sanson, The tooth of perfection: Functional and spatial constraints on mammalian tooth shape. *Biol. J. Linn. Soc.* **78**, 173–191 (2003).

26. G. P. Wilson, A. R. Evans, I. J. Corfe, P. D. Smits, M. Fortelius, J. Jernvall, Adaptive radiation of multituberculate mammals before the extinction of dinosaurs. *Nature* **483**, 457–460 (2012).

27. K. M. Melstrom, R. B. Irmis, Repeated evolution of herbivorous crocodyliforms during the age of dinosaurs. *Curr. Biol.* **29**, 2389–2395.e3 (2019).

28. T. A. Püschel, J. Marcé-Nogué, J. T. Gladman, R. Bobe, W. I. Sellers, Inferring locomotor behaviours in Miocene New World monkeys using finite element analysis, geometric morphometrics and machine-learning classification techniques applied to talar morphology. *J. R. Soc. Interface* **15**, 20180520 (2018).

29. T. A. Püschel, J. Marcé-Nogué, J. Gladman, B. A. Patel, S. Almécija, W. I. Sellers, Getting its feet on the ground: Elucidating *Paralouatta*'s semi-terrestriality using the virtual morpho-functional toolbox. *Front. Earth Sci.* **8**, 15 (2020).

30. J. Marcé-Nogué, S. de Esteban-Trivigno, C. Escrig, L. Gil, Accounting for differences in element size and homogeneity when comparing finite element models: Armadillos as a case study. *Palaeontol. Electron.* **19**, 1–22 (2016).

31. G. Sanson, The biomechanics of browsing and grazing. *Am. J. Bot.* **93**, 1531–1545 (2006).

32. A. Herrel, B. Vanhooydonck, R. Van Damme, Omnivory in lacertid lizards: Adaptive evolution or constraint? *J. Evol. Biol.* **17**, 974–984 (2004).

33. D. E. Winkler, E. Schulz-Kornas, T. M. Kaiser, T. Tutken, Dental microwear texture reflects dietary tendencies in extant Lepidosauria despite their limited use of oral food processing. *Proc. R. Soc. B* **286**, 20190544 (2019).

34. M. D. D'Emic, P. M. O'Connor, T. R. Pascucci, J. N. Gavras, E. Mardakhayava, E. K. Lund, Evolution of high tooth replacement rates in theropod dinosaurs. *PLOS ONE* **14**, e0224734 (2019).

35. S. B. Crofts, S. M. Smith, P. S. L. Anderson, Beyond description: The many facets of dental biomechanics. *Integr. Comp. Biol.* **60**, 594–607 (2020).

36. R. A. Thulborn, Tooth wear and jaw action in the Triassic ornithischian dinosaur *Fabrosaums*. *J. Zool.* **164**, 165–179 (1971).

37. C. E. Gow, J. W. Kitching, M. A. Raath, Skulls of the prosauropod dinosaur M*assospondylus carinatus* Owen in the collections of the Bernard Price Institute for Palaeontological Research. *Palaeontol. Afr.* **27**, 45–58 (1990).

38. P. M. Galton, Cranial anatomy of the prosauropod dinosaur *Plateosaurus* from the Knollenmergel (Middle Keuper, Upper Triassic) of Germany. *Geologica et Palaeontologica* **19**, 119–159 (1985).

39. K. E. J. Chapelle, P. M. Barrett, J. Botha, J. N. Choiniere, *Ngwevu intloko*: A new early sauropodomorph dinosaur from the Lower Jurassic Elliot Formation of South Africa and comments on cranial ontogeny in *Massospondylus carinatus*. *PeerJ* **7**, e7240 (2019).

40. A. Ballell, E. J. Rayfield, M. J. Benton, Osteological redescription of the Late Triassic sauropodomorph dinosaur *Thecodontosaurus antiquus* based on new material from Tytherington, southwestern England. *J. Vertebr. Paleontol.* **40**, e1770774 (2020).

41. M. G. Becerra, D. Pol, C. A. Marsicano, O. W. M. Rauhut, The dentition of Manidens condorensis (Ornithischia; Heterodontosauridae) from the Jurassic Cañadón Asfalto formation of patagonia: Morphology, heterodonty and the use of statistical methods for identifying isolated teeth. *Hist. Biol.* **26**, 480–492 (2014).

42. D. C. D'Amore, R. J. Blumenschine, Komodo monitor (*Varanus komodoensis*) feeding behavior and dental function reflected through tooth marks on bone surfaces, and the application to ziphodont paleobiology. *Paleobiology* **35**, 525–552 (2009).

43. W. L. Abler, The serrated teeth of tyrannosaurid dinosaurs, and biting structures in other animals. *Paleobiology* **18**, 161–183 (1992).

44. A. R. Evans, G. D. Sanson, The effect of tooth shape on the breakdown of insects. *J. Zool.* **246**, 391–400 (1998).

45. A. R. Evans, G. P. Wilson, M. Fortelius, J. Jernvall, High-level similarity of dentitions in carnivorans and rodents. *Nature* **445**, 78–81 (2007).

46. P. M. Barrett, P. Upchurch, The evolution of feeding mechanisms in early sauropodomorph dinosaurs. *Spec. Pap. Palaeontol.* **77**, 91–112 (2007).

47. D. J. Button, P. M. Barrett, E. J. Rayfield, Comparative cranial myology and biomechanics of *Plateosaurus* and *Camarasaurus* and evolution of the sauropord feeding apparatus. *Palaeontology* **59**, 887–913 (2016).

48. D. Pol, O. W. M. Rauhut, M. Becerra, A Middle Jurassic heterodontosaurid dinosaur from Patagonia and the evolution of heterodontosaurids. *Naturwissenschaften* **98**, 369–379 (2011).

49. M. G. Becerra, D. Pol, J. A. Whitlock, L. B. Porro, Tooth replacement *in Manidens condorensis*: Baseline study to address the replacement pattern in dentitions of early ornithischians. *Pap. Palaeontol.* **7**, 1167–1193 (2021).

50. S. L. Brusatte, S. J. Nesbitt, R. B. Irmis, R. J. Butler, M. J. Benton, M. A. Norell, The origin and early radiation of dinosaurs. *Earth Sci. Rev.* **101**, 68–100 (2010).

51. M. J. Benton, Dinosaur success in the Triassic: A noncompetitive ecological model. *Q. Rev. Biol.* **58**, 29–55 (1983).

52. S. L. Brusatte, M. J. Benton, M. Ruta, G. T. Lloyd, Superiority, competition, and opportunism in the evolutionary radiation of dinosaurs. *Science* **321**, 1485–1488 (2008).

53. R. B. Sookias, R. J. Butler, R. B. J. Benson, Rise of dinosaurs reveals major body-size transitions are driven by passive processes of trait evolution. *Proc. R. Soc. B* **279**, 2180–2187 (2012).

54. R. B. Irmis, Evaluating hypotheses for the early diversification of dinosaurs. *Earth Environ. Sci. Trans. R. Soc. Edinb.* **101**, 397–426 (2011).

55. A. W. Crompton, J. Attridge, Masticatory apparatus of the larger herbivores during Late Triassic and early Jurassic times, in *The Beginning of the Age of Dinosaurs*, K. Padian, Ed. (Cambridge Univ. Press, 1986), pp. 223–236.

56. R. T. Müller, J. D. Ferreira, F. A. Pretto, M. Bronzati, L. Kerber, The endocranial anatomy of *Buriolestes schultzi* (Dinosauria: Saurischia) and the early evolution of brain tissues in sauropodomorph dinosaurs. *J. Anat.* **238**, 809–827 (2021).

57. M. Bronzati, O. W. M. Rauhut, J. S. Bittencourt, M. C. Langer, Endocast of the Late Triassic (Carnian) dinosaur *Saturnalia tupiniquim*: Implications for the evolution of brain tissue in Sauropodomorpha. *Sci. Rep.* **7**, 11931 (2017).

58. A. Ballell, J. L. King, J. M. Neenan, E. J. Rayfield, M. J. Benton, The braincase, brain and palaeobiology of the basal sauropodomorph dinosaur *Thecodontosaurus antiquus*. *Zool. J. Linn. Soc.* **193**, 541–562 (2021).

59. P. C. Sereno, The evolution of dinosaurs. *Science* **284**, 2137–2147 (1999).

60. R. J. Butler, P. Upchurch, D. B. Norman, The phylogeny of the ornithischian dinosaurs. *J. Syst. Palaeontol.* **6**, 1–40 (2008).

61. L. Sciscio, F. Knoll, E. M. Bordy, M. O. de Kock, R. Redelstorff, Digital reconstruction of the mandible of an adult *Lesothosaurus diagnosticus* with insight into the tooth replacement process and diet. *PeerJ* **5**, e3054 (2017).

62. R. B. Irmis, W. G. Parker, S. J. Nesbitt, J. Liu, Early ornithischian dinosaurs: The Triassic record. *Hist. Biol.* **19**, 3–22 (2007).

63. D. B. Norman, D. B. Weishampel, "Feeding mechanisms in some small herbivorous dinosaurs: Processes and patterns" in *Biomechanics in Evolution*, M. V. Rayner, R. J. Wootton, Eds. (Cambridge Univ. Press, 1991), pp. 161–182.

64. D. B. Norman, A. W. Crompton, R. J. Butler, L. B. Porro, A. J. Charig, The Lower Jurassic ornithischian dinosaur *Heterodontosaurus tucki* Crompton & Charig, 1962: Cranial anatomy, functional morphology, taxonomy, and relationships. *Zool. J. Linn. Soc.* **163**, 182–276 (2011).

65. M. G. Becerra, D. Pol, G. E. Rossner, O. W. M. Rauhut, Heterodonty and double occlusion in *Manidens condorensis*: A unique adaptation in an Early Jurassic ornithischian improving masticatory efficiency. *Sci. Nat.* **105**, 41 (2018).

66. R. J. Butler, L. B. Porro, D. B. Norman, A juvenile skull of the primitive ornithischian dinosaur *Heterodontosaurus tucki* from the 'Stormberg' of southern Africa. *J. Vertebr. Paleontol.* **28**, 702–711 (2008).

67. M. C. Langer, M. D. Ezcurra, J. S. Bittencourt, F. E. Novas, The origin and early evolution of dinosaurs. *Biol. Rev.* **85**, 55–110 (2010).

68. R. S. Gilmore, R. P. Pollack, J. L. Katz, Elastic properties of bovine dentine and enamel. *Arch. Oral Biol.* **15**, 787–796 (1970).

69. E. R. Dumont, I. R. Grosse, G. J. Slater, Requirements for comparing the performance of finite element models of biological structures. *J. Theor. Biol.* **256**, 96–103 (2009).

70. E. J. Rayfield, Finite element analysis and understanding the biomechanics and evolution of living and fossil organisms. *Annu. Rev. Earth Planet. Sci.* **35**, 541–576 (2007).

71. R Core Team, *R: A Language and Environment for Statistical Computing* (R Foundation for Statistical Computing, 2021).

72. A. Ballell, H. G. Ferrón, Biomechanical insights into the dentition of megatooth sharks (Lamniformes: Otodontidae). *Sci. Rep.* **11**, 1232 (2021).

73. J. Marcé-Nogué, S. de Esteban-Trivigno, T. A. Püschel, J. Fortuny, The intervals method: A new approach to analyse finite element outputs using multivariate statistics. *PeerJ* **5**, e3793 (2017).

74. T. Guillerme, dispRity: A modular R package for measuring disparity. *Methods Ecol. Evol.* **9**, 1755–1763 (2018).

75. D. F. Wiley, N. Amenta, D. A. Alcantara, D. Ghosh, Y. J. Kil, E. Delson, W. Harcourt-Smith, K. St. John, F. J. Rohlf, B. Hamann, Evolutionary morphing. *Proc. IEEE Vis.* **2005**, 431–438 (2005).

76. L. Botton-Divet, R. Cornette, A. C. Fabre, A. Herrel, A. Houssaye, Morphological analysis of long bones in semi-aquatic mustelids and their terrestrial relatives. *Integr. Comp. Biol.* **56**, 1298–1309 (2016).

77. S. Schlager, G. Jefferis, D. Ian, Morpho: Calculations and visualisations related to Geometric Morphometrics. *R package* **2** 9 (2013).

78. P. Gunz, P. Mitteroecker, Semilandmarks: A method for quantifying curves and surfaces. *Hystrix* **24**, 103–109 (2013).

79. P. Gunz, P. Mitteroecker, F. L. Bookstein, Semilandmarks in three dimensions, in *Modern Morphometrics in Physical Anthropology*, D. E. Slice, Ed. (Springer, 2005), pp. 73–98.

80. D. C. Adams, E. Otárola-Castillo, geomorph: An R package for the collection and analysis of geometric morphometric shape data. *Methods Eco. Evol.* **4**, 393–399 (2013).

81. D. C. Adams, A generalized K statistic for estimating phylogenetic signal from shape and other high-dimensional multivariate data. *Syst. Biol.* **63**, 685–697 (2014).

82. D. C. Adams, A method for assessing phylogenetic least squares models for shape and other high-dimensional multivariate data. *Evolution* **68**, 2675–2688 (2014).

83. Ø. Hammer, D. A. Harper, P. D. Ryan, PAST: Paleontological statistics software package for education and data analysis. *Palaeontol. Electron.* **4**, 4 (2001).

84. M. Kuhn, The caret package. *J. Stat. Softw.* **28**, 1–26 (2008).

85. M. Kuhn, K. Johnson, *Applied Predictive Modelling* (Springer, 2013).

86. D. Pol, A. Otero, C. Apaldetti, R. N. Martínez, Triassic sauropodomorph dinosaurs from South America: The origin and diversification of dinosaur dominated herbivorous faunas. *J. South Am. Earth Sci.* **107**, 1031452 (2021).

87. M. G. Baron, D. B. Norman, P. M. Barrett, Postcranial anatomy of *Lesothosaurus diagnosticus* (Dinosauria: Ornithischia) from the Lower Jurassic of southern Africa: Implications for basal ornithischian taxonomy and systematics. *Zool. J. Linn. Soc.* **179**, 125–168 (2017).

88. D. Madzia, V. M. Arbour, C. A. Boyd, A. A. Farke, P. Cruzado-Caballero, D. C. Evans, The phylogenetic nomenclature of ornithischian dinosaurs. *PeerJ* **9**, e12362 (2021).

89. P. C. Sereno, F. E. Novas, The complete skull and skeleton of an early dinosaur. *Science* **258**, 1137–1140 (1992).

90. R. T. Müller, M. C. Langer, M. Bronzati, C. P. Pacheco, S. F. Cabreira, Sérgio Dias-Da-Silva, Early evolution of sauropodomorphs: Anatomy and phylogenetic relationships of a remarkably well-preserved dinosaur from the Upper Triassic of southern Brazil. *Zool. J. Linn. Soc.* **184**, 1187–1248 (2018).

91. M. C. Langer, B. W. McPhee, J. C. D. Marsola, L. Roberto-da-Silva, S. F. Cabreira, Anatomy of the dinosaur *Pampadromaeus barberenai* (Saurischia — Sauropodomorpha) from the Late Triassic Santa Maria Formation of southern Brazil. *PLOS ONE* **14**, e0212543 (2019).

92. M. C. Langer, F. Abdala, M. Richter, M. J. Benton, A sauropodomorph dinosaur from the Upper Triassic (Carnian) of southern Brazil. *C. R. Acad. Sci. Ser. II* **329**, 511–517 (1999).

93. M. J. Benton, L. Juul, G. W. Storrs, P. M. Galton, Anatomy and systematics of the prosauropod dinosaur *Thecodontosaurus antiquus* from the Upper Triassic of southwest England. *J. Vertebr. Paleontol.* **20**, 77–108 (2000).

94. A. Prieto-Márquez, M. A. Norell, Redescription of a nearly complete skull of *Plateosaurus* (Dinosauria: Sauropodomorpha) from the Late Triassic of Trossingen (Germany). *Am. Mus. Novit.* **3727**, 1–58 (2011).

95. J. N. Lallensack, E. M. Teschner, B. Pabst, P. M. Sander, New skulls of the basal sauropodomorph *Plateosaurus trossingensis* from Frick, Switzerland: Is there more than one species? *Acta Palaeontol. Pol.* **66**, 1–28 (2021).

96. K. E. J. Chapelle, J. N. Choiniere, A revised cranial description of *Massospondylus carinatus* Owen (Dinosauria: Sauropodomorpha) based on computed tomographic scans and a review of cranial characters for basal Sauropodomorpha. *PeerJ* **6**, e4224 (2018).

97. R. A. Pyron, F. T. Burbrink, J. J. Wiens, A phylogeny and revised classification of Squamata, including 4161 species of lizards and snakes. *BMC Evol. Biol.* **13**, 93 (2013).

98. J. P. Rio, P. D. Mannion, Phylogenetic analysis of a new morphological dataset elucidates the evolutionary history of Crocodylia and resolves the long-standing gharial problem. *PeerJ* **9**, e12094 (2021).

99. D. I. Whiteside, J. E. A. Marshall, The age, fauna and palaeoenvironment of the Late Triassic fissure deposits of Tytherington, South Gloucestershire, UK. *Geol. Mag.* **145**, 105–147 (2008).

100. M. C. Langer, J. Ramezani, A. A. S. Da Rosa, U-Pb age constraints on dinosaur rise from south Brazil. *Gondw. Res.* **57**, 133–140 (2018).

101. M. J. Benton, P. C. J. Donoghue, R. J. Asher, M. Friedman, T. J. Near, J. Vinther, Constraints on the timescale of animal evolutionary history. *Palaeontol. Electron.* **18**, 1FC (2015).

102. T. R. Simões, M. W. Caldwell, M. Tałanda, M. Bernardi, A. Palci, O. Vernygora, F. Bernardini, L. Mancini, R. L. Nydam, The origin of squamates revealed by a Middle Triassic lizard from the Italian Alps. *Nature* **557**, 706–709 (2018).

103. D. W. Bapst, paleotree: An R package for paleontological and phylogenetic analyses of evolution. *Methods Ecol. Evol.* **3**, 803–807 (2012).

104. W. Y. Loh, Classification and regression *trees. Data Min. Knowl. Discov.* **1**, 14–23 (2011).

105. R. A. Fisher, The use of multiple measurements in taxonomic problems. *Ann. Hum. Genet.* **7**, 179–188 (1936).

106. B. L. Welch, Note on discriminant functions. *Biometrika* **31**, 218–220 (1936).

107. T. Hastie, R. Tibshirani, Discriminant analysis by Gaussian mixtures. *J. R. Stat. Soc. B* **58**, 155–176 (1996).

108. V. N. Vapnik, *The Nature of Statistical Learning Theory* (Springer, 2000).

109. T. M. Cover, P. E. Hart, Nearest neighbor pattern classification. *IEEE Trans. Inf. Theory* **13**, 21–27 (1967).

110. P. Domingos, M. Pazzani, On the optimality of the simple Bayesian classifier under zero-one loss. *Mach. Learn.* **29**, 103–130 (1997).

111. L. Breiman, Random forests. *Mach. Learn.* **45**, 5–32 (2001).

112. M. Titterington, Neural networks. *Wiley Interdiscip. Rev. Comput. Stat.* **2**, 1–8 (2010).

113. W. E. Cooper, L. J. Vitt, Distribution, extent, and evolution of plant consumption by lizards. *J. Zool.* **257**, 487–517 (2002).

114. S. Meiri, Traits of lizards of the world: Variation around a successful evolutionary design. *Glob. Ecol. Biogeogr.* **27**, 1168–1172 (2018).

115. L. Luiselli, E. Filippi, M. Capula, Geographic variation in diet composition of the grass snake (*Natrix natrix*) along the mainland and an island of Italy: The effects of habitat type and interference with potential competitors. *Herpetol. J.* **15**, 221–230 (2005).

116. S. Bhupathy, C. Ramesh, A. Bahuguna, Feeding habits of Indian rock pythons in Keoladeo National Park, Bharatpur, India. *Herpetol. J.* **24**, 59–64 (2014).

117. J. B. Thorbjarnarson, Notes on the feeding behavior of the gharial (*Gavialis gangeticus*) under seminatural conditions. *J. Herpetol.* **24**, 99–100 (1990).

118. O. S. G. Pauwels, V. Mamonekene, P. Dumont, W. R. Branch, M. Burger, S. Lavoué, Diet records for C*rocodylus cataphractus* (Reptilia: Crocodylidae) at Lake Divangui, Ogooué-Maritime Province, south-western Gabon. *Hamadryad* **27**, 200–204 (2003).

119. A. Ősi, P. M. Barrett, Dental wear and oral food processing in *Caiman latirostris*: Analogue for fossil crocodylians with crushing teeth. *Neues Jahrb. fur Geol. Palaontol. - Abh.* **261**, 201–207 (2011).

120. E. C. Herbst, S. Lautenschlager, D. Bastiaans, F. Miedema, T. M. Scheyer, Modeling tooth enamel in FEA comparisons of skulls: Comparing common simplifications with biologically realistic models. *iScience* **24**, 103182 (2021).
